# A novel specific PERK activator reduces toxicity and extends survival in Huntington's disease models

**DOI:** 10.1038/s41598-020-63899-4

**Published:** 2020-04-23

**Authors:** Javier Ganz, Talya Shacham, Maria Kramer, Marina Shenkman, Hagit Eiger, Nitai Weinberg, Ori Iancovici, Somnath Roy, Luba Simhaev, Benny Da’adoosh, Hamutal Engel, Nisim Perets, Yael Barhum, Moshe Portnoy, Daniel Offen, Gerardo Z. Lederkremer

**Affiliations:** 1 Department of Human Molecular Genetics and Biochemistry, Felsenstein Medical Research Center, Sackler School of Medicine, Tel Aviv University, Tel Aviv, 69978 Israel; 2 Sagol School of Neuroscience, Tel Aviv University, Tel Aviv, 69978 Israel; 3 School of Molecular Cell Biology and Biotechnology, George Wise Faculty of Life Sciences, Tel Aviv University, Tel Aviv, 69978 Israel; 4 Blavatnik Center for Drug Discovery, George Wise Faculty of Life Sciences, Tel Aviv University, Tel Aviv, 69978 Israel; 5 School of Chemistry, Raymond and Beverly Sackler Faculty of Exact Sciences, Tel Aviv University, Tel Aviv, 69978 Israel

**Keywords:** Mechanisms of disease, Huntington's disease

## Abstract

One of the pathways of the unfolded protein response, initiated by PKR-like endoplasmic reticulum kinase (PERK), is key to neuronal homeostasis in neurodegenerative diseases. PERK pathway activation is usually accomplished by inhibiting eIF2α-P dephosphorylation, after its phosphorylation by PERK. Less tried is an approach involving direct PERK activation without compromising long-term recovery of eIF2α function by dephosphorylation. Here we show major improvement in cellular (ST*Hdh*^*Q111/111*^) and mouse (R6/2) Huntington's disease (HD) models using a potent small molecule PERK activator that we developed, MK-28. MK-28 showed PERK selectivity *in vitro* on a 391-kinase panel and rescued cells (but not PERK−/− cells) from ER stress-induced apoptosis. Cells were also rescued by the commercial PERK activator CCT020312 but MK-28 was significantly more potent. Computational docking suggested MK-28 interaction with the PERK activation loop. MK-28 exhibited remarkable pharmacokinetic properties and high BBB penetration in mice. Transient subcutaneous delivery of MK-28 significantly improved motor and executive functions and delayed death onset in R6/2 mice, showing no toxicity. Therefore, PERK activation can treat a most aggressive HD model, suggesting a possible approach for HD therapy and worth exploring for other neurodegenerative disorders.

## Introduction

HD is a neurodegenerative disease arising from an expanded CAG repeat in the exon 1 of the huntingtin gene, which translates into a polyglutamine (polyQ) tract in the huntingtin (Htt) protein ^1,2^. HD is a genetic, autosomal dominant disease with late onset and progressive motor dysfunction, cognitive decline and behavioral abnormalities. In addition to these, other systemic impairments such as weight loss, muscle wasting and glucose regulation impairment were also reported^2^. The expansion of the polyQ repeats causes mutant Htt (mHtt) to aggregate in HD tissues when it includes above 35 glutamine residues, with a consequent induction of cellular stress, toxicity and cell death especially in the brain striatum and extending later to the cortex. This reflects progressively in the deterioration of the individual's biological functions ^3,4^. Although several therapeutic approaches are currently being pursued, including ongoing clinical trials for lowering mHtt levels using antisense oligonucleotides (ASOs), there is currently no effective treatment for HD^5^.

One of the important consequences of the gradual accumulation of misfolded mHtt is its inhibition of ER-associated degradation (ERAD), causing endoplasmic reticulum (ER) stress and induction of a conserved stress response known as the unfolded protein response (UPR) ^6–12^. The function of the UPR is to either re-establish cellular homeostasis or, if this fails, to trigger cell death in order to prevent further accumulation of damaged, non-functional cells. We have previously reported that there is a strong ER stress induction and UPR activation in striatal neurons expressing mHtt^13^. Importantly, striatal neurons are the first to degenerate in HD patients. In mammals, the UPR includes three signaling pathways initiated by their sensors, the ER-resident transmembrane proteins PKR-like endoplasmic reticulum kinase (PERK), activating transcription factor-6 (ATF6), and inositol-requiring enzyme-1 (IRE1)^14^. Activation of PERK induces the phosphorylation of the eukaryotic translation initiation factor 2-alpha (eIF2α) and of transcription factor erythroid 2-related factor 2 (NRF2)^15^. NRF2 phosphorylation initiates a cell-protective anti-oxidant response, while eIF2α phosphorylation causes a transient global suppression of protein translation, reducing ER protein load and stress ^16,17^. In addition, phosphorylated eIF2α enhances the translation of activating transcription factor-4 (ATF4), which upregulates beneficial UPR target genes required for autophagy, antioxidant response, and amino acid metabolism^18^. Direct PERK activation was never explored until recently, when neuroprotection by compound CCT020312 was reported in models of tauopathies^19^. These protein aggregation disorders share many common pathophysiological pathways with HD, particularly high ER stress status, where modulation of PERK could result in beneficial effects. PERK modulation has not been evaluated so far in animal models of HD. We had shown that the PERK pathway is strongly downregulated in striatal neurons compared to other cell types and brain regions in WT mice and is upregulated in HD^13^. We initially interpreted this activation of the PERK pathway as a possible cause of cellular dysfunction and toxicity. However, the results that we report here suggest that PERK activation in response to mHtt expression is an insufficient cellular attempt to restore homeostasis. Here we synthesized and tested novel structurally-related derivatives of a PERK modulator, finding several compounds that reduced mHtt cytotoxicity in a cellular HD model (a murine striatal cell line with knock-in of a full-length polyQ-expanded mHtt (ST*Hdh*^Q111/111^)^20^. The effective compounds are predicted to interact with the PERK activation loop. For the most effective one, MK-28, we determined that it is a potent and selective PERK activator that strongly reduces cytotoxicity and improves motor and executive functions, extending the lifespan of the most aggressive HD mouse model, the R6/2 with 160 CAG repeats.

## Results

### MK-28 is non-toxic and neuroprotective in HD model striatal neurons

**Figure 1:**
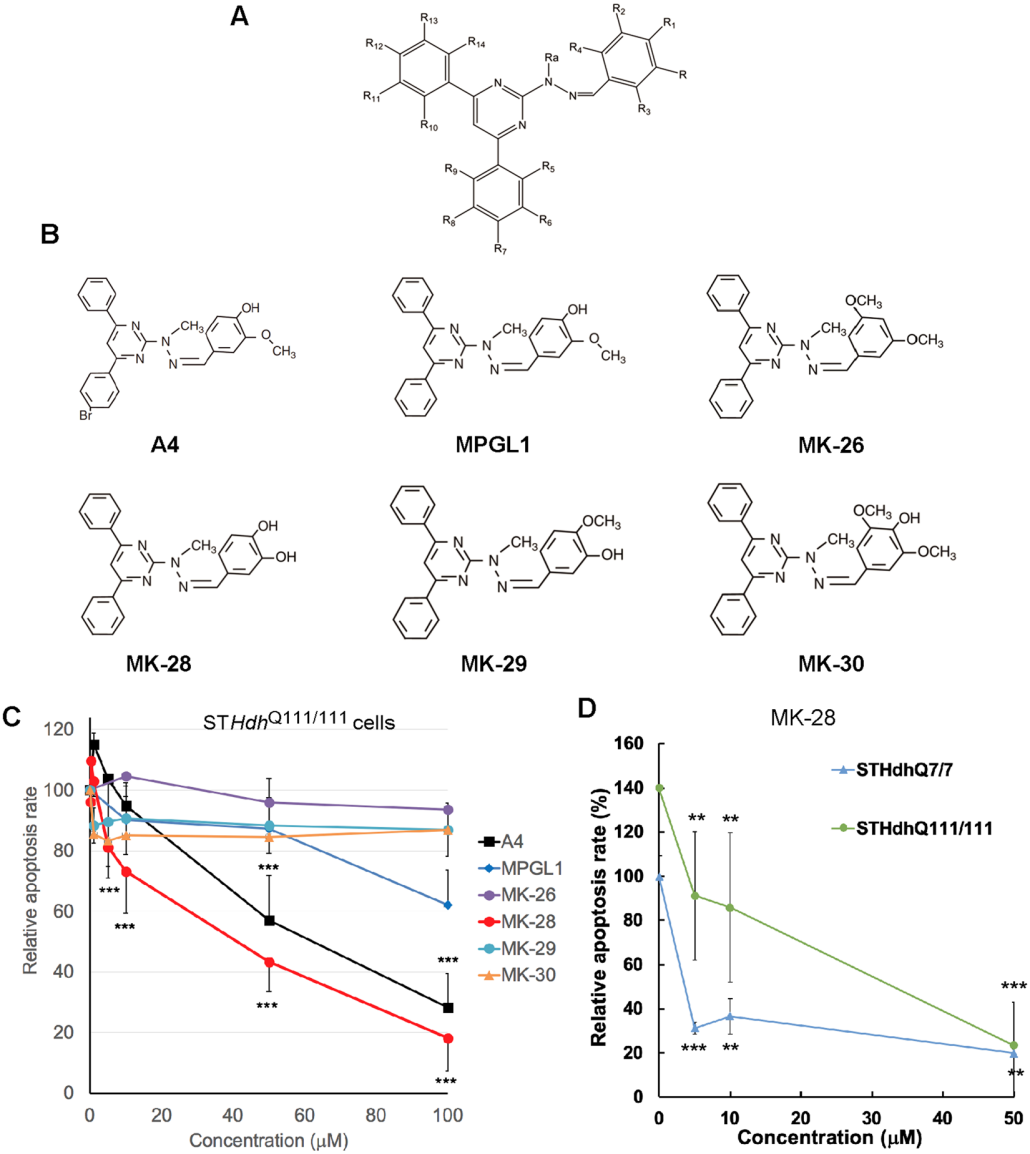
MK-28 rescues cells from ER stress-induced apoptosis. (**A)** General structural formula of the evaluated compounds. (**B)** Structures of A4 (Chembridge database) and novel derivatives. (**C)** Apoptosis rescue experiment using the murine striatal cell line expressing full-length polyQ-expanded Htt (ST*Hdh*^Q111/111^) incubated with the ER stress-inducer tunicamycin, shows that MK-28 and to a lesser extent A4 and MPGL1 are able to rescue the cells from apoptosis. Apoptosis was measured after 48 h of Tun exposure and PERK modulators were tested at different concentrations (0–100 μM). Results are expressed as average relative apoptosis rate ± SD of 3 independent experiments; apoptosis in the presence of tunicamycin and in the absence of other compounds was normalized to 100 to allow comparison between experiments. P values MK-28 vs. only Tun: 5 μM = 6 × 10^–5^, 50 μM = 1 × 10^–9^, 100 μM = 5 × 10^–13^; A4 vs. only Tun: 50 μM = 8 × 10^–5^, 100 μM = 0.001. (**D)** Rescue of ST*Hdh*^Q111/111^ cells compared to ST*Hdh*^Q7/7^ cells by MK-28. The graph shows average relative apoptosis rate ± SD of 3 independent experiments. P values vs. only Tun: ST*Hdh*^Q7/7^ cells: 5 μM = 2 × 10^–5^, 10 μM = 0.0002, 50 μM = 0.004, ST*Hdh*^111/111^ cells: 5 μM = 0.006, 10 μM = 0.008, 50 μM = 8 × 10^–6^.

We had previously reported protective effects of A4, a compound that reversed the toxicity of mHtt expression in a striatal neuron cell line^13^. A4 had been found in a screen for PERK inhibitors^21^. We now synthesized small molecule derivatives of the mother compound A4 and tested their ability to prevent cytotoxicity induced by pathogenic mHtt (Fig. 1). The derivatives were closely related analogues, in order to evaluate the effect of alternative substitution group patterns (Fig. 1A,B). We used the stable murine striatal cell line expressing full-length polyQ-expanded mHtt (ST*Hdh*^Q111/111^) for the rescue experiment. Although as an immortalized cell line these cells show low levels of apoptosis, we had observed that they are much more sensitive than their counterpart expressing WT Htt (ST*Hdh*^Q7/7^ cells) and than other cell types to the ER stressor and apoptosis inducer tunicamycin (Tun)^13^. We incubated ST*Hdh*^Q111/111^ cells with Tun and in parallel with increasing concentrations of the different PERK modulators. Apoptosis was measured after 48 h of Tun exposure and data analysis showed a strong cellular protection by MK-28 in a dose-dependent manner, reducing apoptosis by 5-fold at 100 μM (Fig. 1C, Supplementary Fig. S1). The protective effect of MK-28 was stronger than that of the mother compound A4 and of the other four derivatives tested (MPGL-1, MK26, MK-29, MK-30). MK-28 was particularly more effective than A4 at low concentrations, reducing apoptosis by 20% at 5 μM compared to no significant effect by A4. The activity appears to be highly dependent on the substitution pattern, with active compounds having an OH in position R1 and an OCH3 or preferably an OH in position R (Fig. 1A). When the effect of MK-28 was compared between ST*Hdh*^Q111/111^ and ST*Hdh*^Q7/7^ cells, a concentration was reached (50 μM) that showed identical levels of apoptosis (Fig. 1D). Therefore, MK-28 compensated the additional toxicity due to the expression of pathogenic huntingtin.

### MK-28 effect on the PERK pathway in striatal cells expressing mHtt

**Figure 2:**
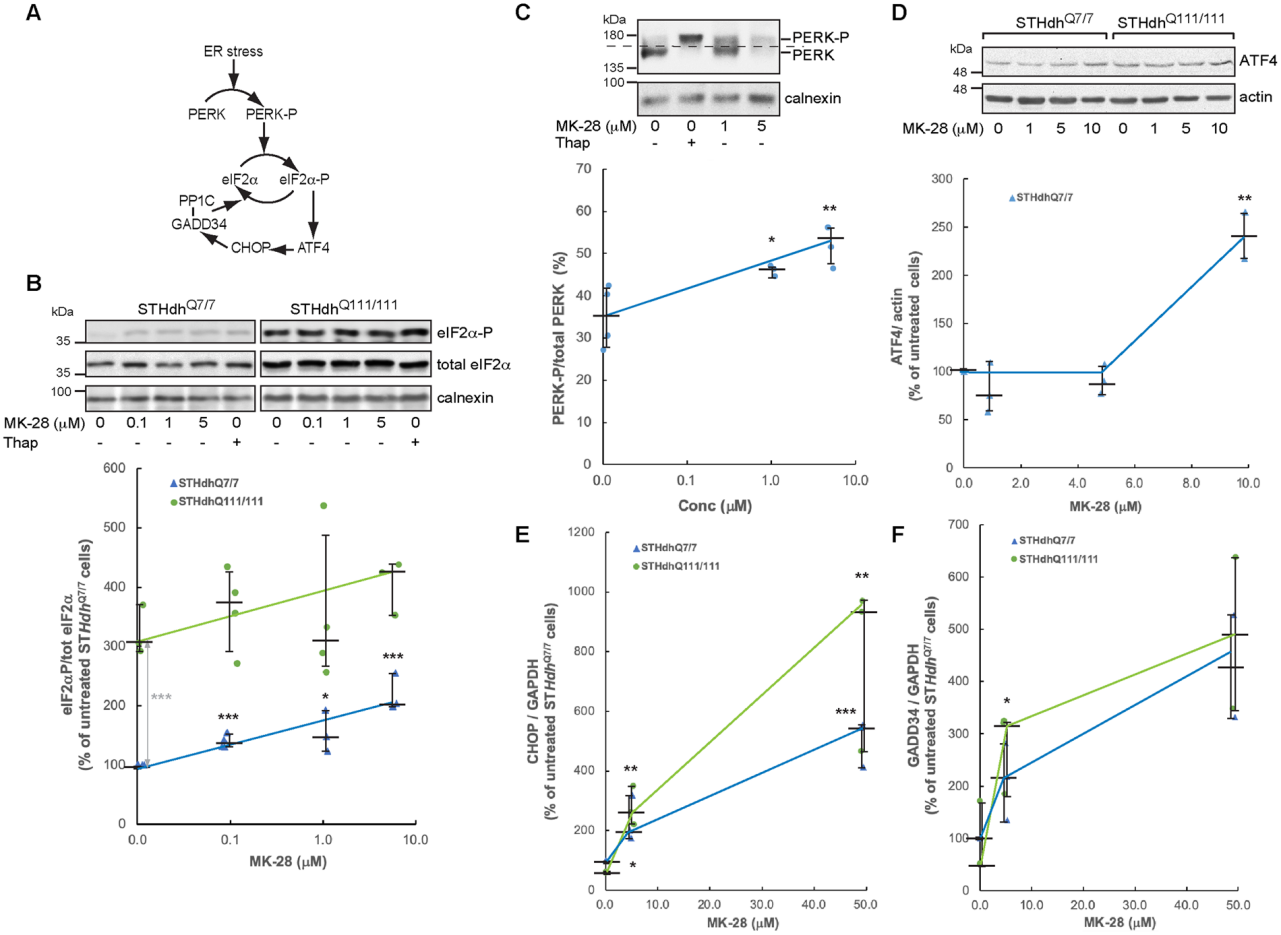
MK-28 is a PERK activator in an HD cellular model. (**A**) Scheme of the PERK pathway. **(B)** Treatment of ST*Hdh*^Q7/7^ and ST*Hdh*^Q111/111^ cells (expressing WT and pathogenic Htt respectively) with MK-28 (0–5 μM) or Thap (2 μg/ml) for 1.5 hours caused an increase of eIF2α-P levels relative to total eIF2α-P for both cell types. Calnexin was used as loading control. See full length blots in Supplementary Fig. S6. In the graph, results are compared to untreated ST*Hdh*^Q7/7^ cells (=100) and expressed as medians +- interquartile range of 4 independent experiments. P values treated vs. untreated in black (ST*Hdh*^Q7/7^ MK-28 0.1 μM vs. untr = 0.0004, ST*Hdh*^Q7/7^ MK-28 1 μM vs. untr = 0.02, ST*Hdh*^Q7/7^ MK-28 5 μM vs. untr = 0.0005) and between the two untreated cell lines in grey (Untr. ST*Hdh*^Q111/111^ vs. untr. ST*Hdh*^Q7/7^ = 0.0001). (**C)** Treatment of MEF cells with MK-28 or Thap (2 μg/ml) for 3 hours caused a shift of PERK consistent with its phosphorylation (PERK-P). The dashed line is for better visualization of the shift. The graph shows an estimation of PERK-P/total PERK (population over the dashed line/total population), expressed as medians +- interquartile range of 4 independent experiments. P values MK-28 1 μM vs. Cntl = 0.02, MK-28 5 μM vs. Cntl = 0.003. (**D)** Similar to (B) but for ATF4, with actin as loading control. Quantified only for ST*Hdh*^Q7/7^. P value MK-28 10 μM vs. untr = 0.009. (**E**,**F)** Cell lysates of ST*Hdh*^Q7/7^ and ST*Hdh*^Q111/111^ cells were subjected to qPCR for CHOP (E) and GADD34 mRNA (F), compared to a housekeeping gene, GAPDH. The graphs show values relative to untreated ST*Hdh*^Q7/7^ cells and are medians +- interquartile range of 3 independent experiments. P values: CHOP: ST*Hdh*^Q7/7^ MK-28 5 μM vs. untr = 0.04, ST*Hdh*^Q7/7^ MK-28 50 μM vs. untr = 0.0009, ST*Hdh*^Q111/111^ MK-28 5 μM vs. untr = 0.005, ST*Hdh*^Q111/111^ MK-28 50 μM vs untr = 0.01, GADD34: ST*Hdh*^Q111/111^ MK-28 5 μM vs. untr = 0.04.

The effect of MK-28 was further tested in ST*Hdh*^Q111/111^ cells, compared to ST*Hdh*^Q7/7^ cells. The cells were treated for 1.5 hours with increasing concentrations of MK-28 and the levels of phosphorylation of the PERK substrate eIF2α (eIF2α-P) (Fig. 2A) were determined. Reflecting their ER stress status^13^, ST*Hdh*^Q111/111^ cells showed three-fold higher levels of eIF2α-P than ST*Hdh*^Q7/7^ cells at baseline with no MK-28 exposure, as we had seen before^13^. MK-28 did not reduce eIF2α-P levels but instead caused an increase for both cell types, similar to that caused by an ER stressor, thapsigargin (Thap). The increase was significant, up to almost 2-fold, for ST*Hdh*^Q7/7^ cells (Fig. 2B). This was unexpected, because the mother compound had been found in a screen for PERK inhibitors. We then looked directly at PERK. This experiment was done with MEF cells, which showed a much higher PERK level than the striatal cells and could therefore be clearly visualized. MK-28 treatment caused a shift of part of the PERK population to a slower migration, consistent with PERK-P and similar to that obtained by cell treatment with Thap (Fig. 2C). The lower signal at 5 μM MK-28 is due to a shift of part of the population to higher MW, possibly dimers and oligomers of activated PERK (Supplementary Fig. S6)). Downstream factors of the PERK pathway were also upregulated, including ATF4, CHOP and GADD34 (Fig. 2D–F). ATF4 protein levels were increased significantly, up to 2.5-fold, in ST*Hdh*^Q7/7^ cells at high MK-28 concentration, whereas there was a large variation in ST*Hdh*^Q111/111^ cells between experiments. CHOP and GADD34 mRNA levels were measured by quantitative real-time PCR (qPCR) and showed a significant increase in both cell types, up to 10- and 5-fold respectively (Fig. 2E,F).

### MK-28 activates PERK *in vitro*

**Figure 3:**
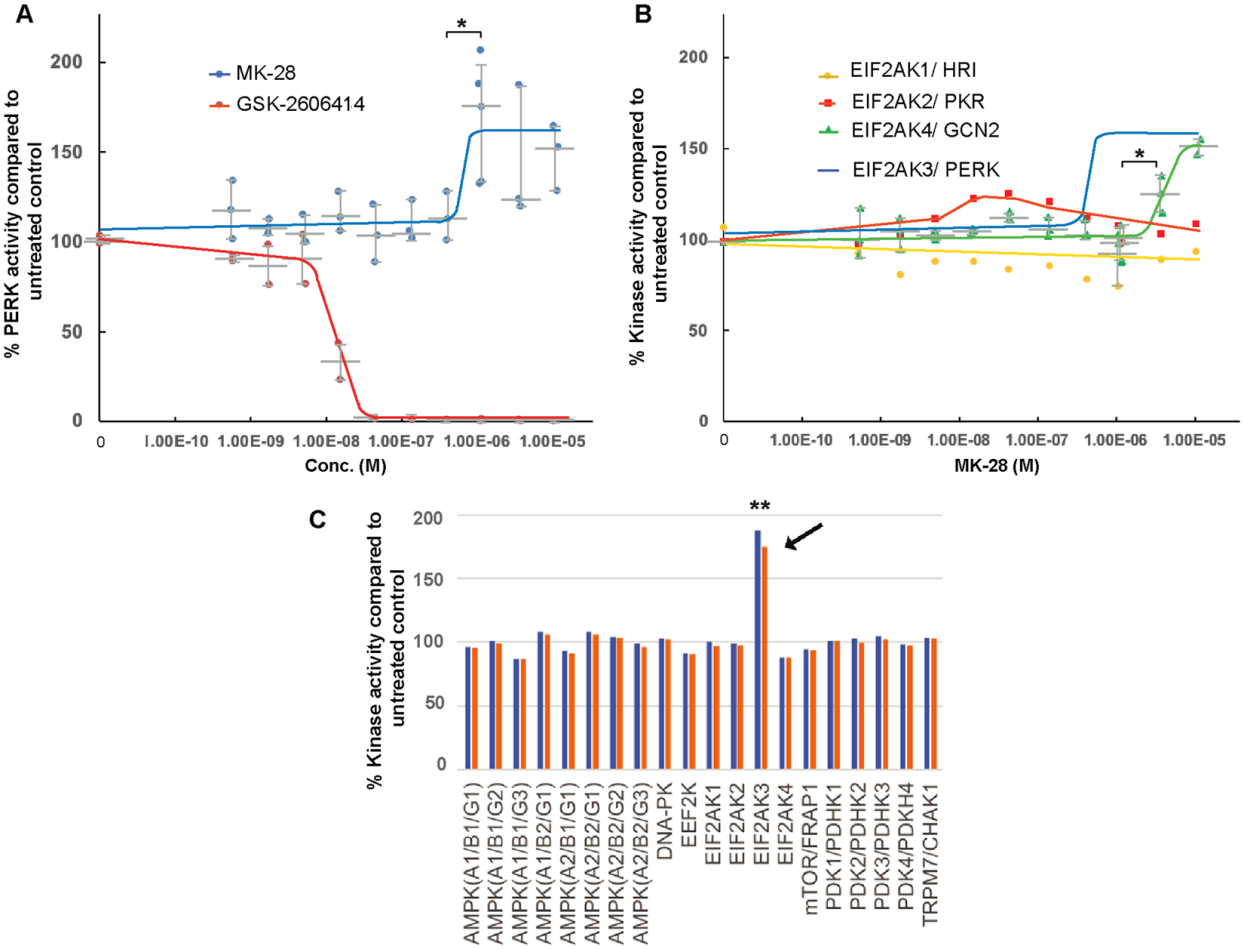
MK-28 selectively activates PERK using purified components *in vitro*. (**A)** MK-28 was tested for its effect on isolated PERK *in vitro*, showing an activating effect starting in the nanomolar range with an EC50 of 490 nM (see Supplementary Fig. S2). It was compared to the PERK inhibitor GSK606414, which exhibited the opposite effect than MK-28, showing strong inhibition. The graph shows PERK activity compared to untreated control (100%) (see Methods), expressed as medians +- interquartile range of 3 independent experiments. P value MK-28 1.11 μM vs. 0.37 μM = 0.04. (**B)** MK-28 revealed an activating effect, but in the micromolar range, on the related EIF2AK4 kinase (GCN2) with an EC50 of 3.5 μM, while no effect was observed for EIF2AK1 or EIF2AK2. Graph as in (A), P value for GCN2, MK-28 3.33 μM vs. 1.11 μM = 0.03. (**C)** MK-28 was tested over a panel of 391 kinases at 1 μM in duplicate tests (red and blue). EIF2AK3 (PERK) was the highest hit of the panel with an activation of 181,4% of the control (black arrow), P value vs. untreated = 0.006. The experiment was performed by Reaction Biology Corp. (see Methods) and only the “Atypical kinase panel” results are shown, including the four eIF2α kinases, (see Supplementary Table S1 for complete results for all 391 kinases).

To understand the mechanism of action of MK-28, the effect was analysed *in vitro* on the activity of isolated PERK (EIF2AK3) (performed at Reaction Biology Corp. (Malvern, PA, USA) using the HotSpot Kinase Assay, see Methods). MK-28 activated PERK, starting in the nanomolar range, with an EC50 of 490 nM (Fig. 3A, Supplementary Fig. S2). The results, especially in the high concentration range, may reflect allosteric effects, which should be further investigated in future studies. As expected, a commercial PERK inhibitor, GSK606414^22^ showed a clear inhibitory effect in this assay. In addition, we also tested the effect of MK-28 on the other three eIF2α kinases. MK-28 had little or no effect on EIF2AK1 (HRI) or EIF2AK2 (PKR), but it activated EIF2AK4 (GCN2), although at almost one order of magnitude higher EC50 than with PERK (3.5 μM) (Fig. 3B and Supplementary Fig. S2). The specificity of MK-28 was evaluated on a panel of 391 kinases, showing the highest hit for PERK activation (EIF2AK3, 81,4% increase over control), with only 3 other hits showing more than 30% increase over control (Fig. 3C, Supplementary Table S1).

**Figure 4:**
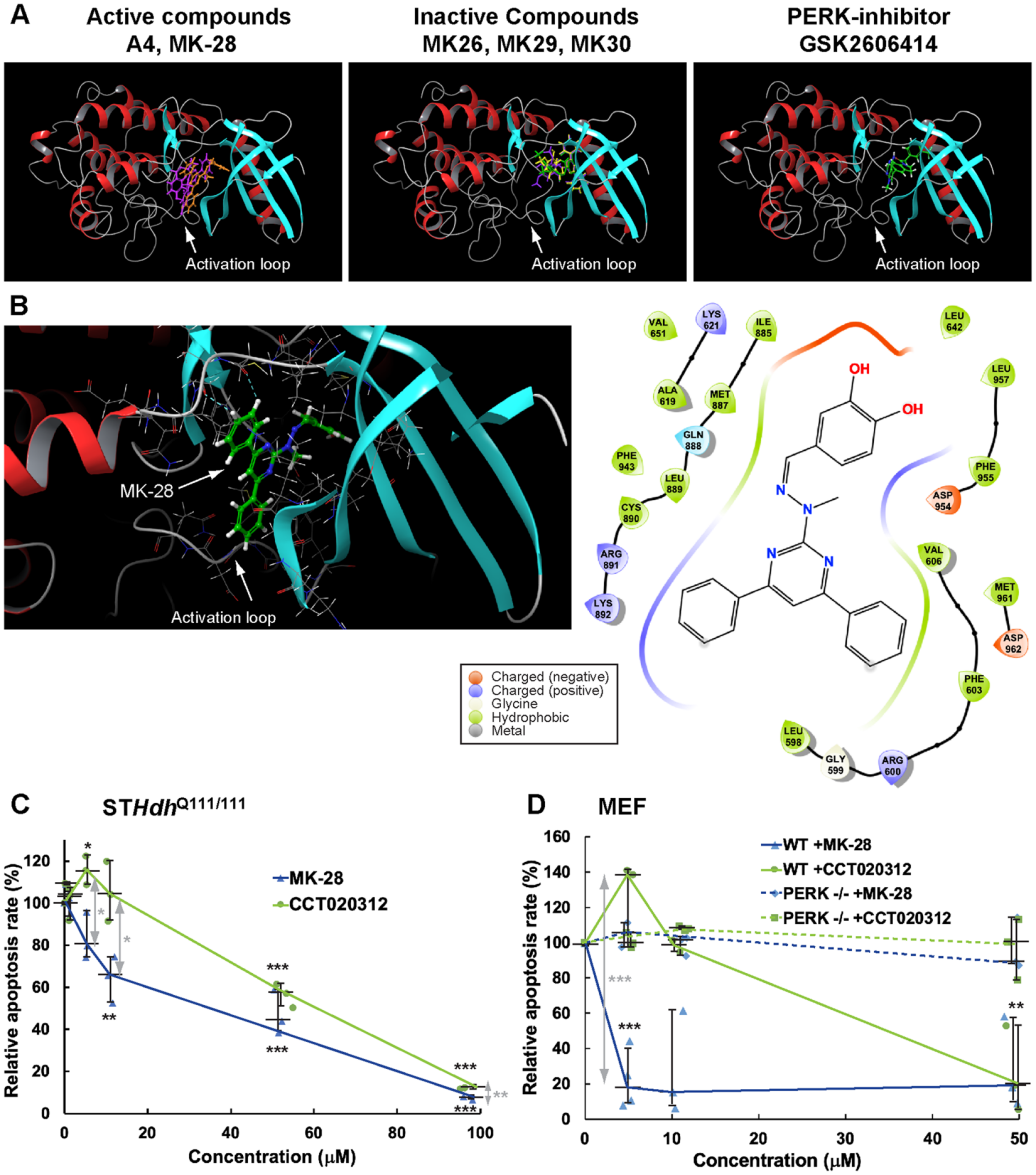
MK-28 is predicted to interact with the PERK activation loop and its cellular protective effect is PERK-dependent and more potent than CCT020312. (**A**) Computer-modeling docking of the synthesized PERK modulators into the PERK structure shows that active compounds (A4 and MK-28) and inactive compounds (MK26, MK29, and MK30) occupy different regions in the binding site of PERK. A crystal structure of the PERK inhibitor GSK2606414 bound to PERK shows that it does not interact with the PERK activation loop (gray), whereas MK-28 and A4 do. Protein is shown as a ribbon diagram with colors according to secondary structure and ligands are shown as sticks. (**B)** Detail of the predicted amino acid interactions of MK-28 with PERK. (**C)** Rescue of ST*Hdh*^Q111/111^ cells by MK-28 compared to the commercial PERK activator CCT020312 from UPR-induced cell death, shows that MK-28 has a more efficient protective effect, especially in the low concentration range. The graph shows medians +- interquartile range of 3 independent experiments for each compound. P values vs. only Tun in black: MK-28 10 μM = 0.005, 50 μM = 0.0009, 100 μM = 4 × 10^–7^; CCT020312 5 μM = 0.02, CCT020312 50 μM = 0.0002, CCT020312 100 μM = 5 × 10^–8^, and between CCT020312 and MK-28 in grey at 5 μM = 0.01, 10 μM = 0.02, 100 μM = 0.005. (**D)** Comparison of rescue between WT and PERK−/− MEF cells by MK-28 and CCT020312 from ER stress-induced cell death, shows that MK-28 is more effective in the low concentration range in WT cells, but fails entirely to protect in PERK−/− cells, indicating high specificity of the cell rescue through PERK. For comparison purposes, apoptosis with only tunicamycin was normalized to 100, as before, although PERK−/− MEFs showed reduced apoptosis levels, about half to two thirds of the WT cells. The graph shows medians +- interquartile range of 3 independent experiments for each compound. P values vs. only Tun in black for WT cells: MK-28 5 μM = 9 × 10^–5^, 50 μM = 0.009 and between CCT020312 and MK-28 in grey at 5 μM = 9 × 10^–5^.

Homology modeling and compound docking may provide an explanation for the activation of PERK, revealing poses for MK-28 and also for A4 that are predicted to interact with the kinase activation loop of PERK (Fig. 4A). Interactions (mainly hydrophobic) were predicted for MK-28 with several residues of the activation loop (Fig. 4B). Interestingly, the compounds that did not protect against cytotoxicity in ST*Hdh*^Q111/111^ cells (Fig. 1) interacted with a region close to the catalytic pocket of PERK. This was true also for the PERK inhibitor (GSK2606414). These compounds showed no poses interacting with the region of the activation loop (Fig. 4A). These results would predict that also A4 might be a PERK activator. We tested this prediction using the *in vitro* kinase activity assay. Indeed, A4 activated PERK (EIF2AK3) in the nanomolar range, although to a small extent (Supplementary Fig. S3).

We then compared the protective effect of MK-28 to that of a commercial PERK activator (CCT020312) on the survival of ST*Hdh*^Q111/111^ cells treated with Tun (Fig. 4C). Over three independent experiments, both compounds protected the ST*Hdh*^Q111/111^ cells similarly at high concentrations (>50 μM), but this was not the case at lower concentrations, where CCT020312 failed to be protective below 10 μM (Fig. 4C). In contrast, MK-28 reduced apoptosis by 40% at 10 μM and was also effective at lower concentrations. The IC50 calculated for inhibition of apoptosis in this experiment is 6.8 μM for MK-28 and 32.4 μM for CCT020312 (Supplementary Fig. S4A). To evaluate whether the cytoprotective activity of MK-28 depends on PERK, we used PERK−/− MEF cells compared to WT MEFs. Both MK-28 and CCT020312 failed to protect the PERK−/− cells, indicating that the protective mechanism is indeed PERK-dependent (Fig. 4D). In addition, the protective effect was stronger in the WT MEFs than in the ST*Hdh*^Q111/111^ cells, with almost an order of magnitude difference in the efficacy of the compounds, IC50 = 1.7 μM for MK-28 and 11.1 μM for CCT020312 (Supplementary Fig. S4B). We also tested whether any of the cytoprotective effect was due to activation of another PERK substrate, NRF2. The mRNA levels of a downstream target of NRF2-P, HMOX1, showed upregulation by MK-28, as expected, in ST*Hdh*^Q7/7^ and ST*Hdh*^Q111/111^ cells (Suppl. Fig. S5A-B). We then performed an experiment similar to that in Fig. 1C, but with the addition of increasing concentrations of ML385, an NRF2 inhibitor, to test whether it would block the beneficial effect of MK-28. ML385 caused some toxicity, as expected, in ST*Hdh*^Q111/111^ cells but did not reverse to any significant extent the cytoprotective effect of MK-28 (Suppl. Fig. S5C-D). Cell protection by MK-28 does not appear to be through NRF2.

### *In vivo* evaluation of MK-28

**Figure 5:**
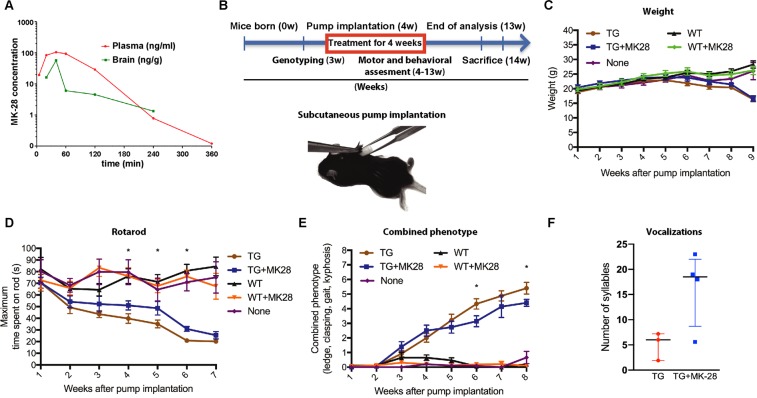
MK-28 exhibits brain penetrance and improves neurological functions in the R6/2 HD mouse model. (A) Pharmacokinetics and BBB penetration analysis show that following 10 mg/kg IP MK-28 injection, a maximum concentration of 105 ng/ml was determined in plasma. MK-28 exhibits good BBB penetrance with a maximum concentration of 57 ng/g. (**B)** Experimental timeline representation in weeks of an experiment comparing R6/2 mice or WT littermates treated with MK-28 or with control vehicle delivered by subcutaneous Alzet osmotic minipumps (n = 13 TG, 13 TG + MK-28, 16 WT, 15 WT + MK-28, 15 None). Below is an illustration of minipump subcutaneous implantation in mice. (**C)** Weight analysis during 9 weeks after treatment initiation. (**D)** Rotarod test shows significant improvement in motor function upon treatment of R6/2 mice with MK-28. TG mice showed a strong motor deficit, which was significantly reduced with MK-28 treatment, continuing after the end of administration of the compound. Significance *p < 0.05 Anova Newman-Keuls post hoc. (**E)** Combined phenotype test (ledge, clasping, gait and kyphosis) shows significant improvement upon treatment of R6/2 mice with MK-28. Significance *p < 0.05. (**F)** Ultrasonic vocalizations of R6/2 mice are improved by treatment with MK-28. TG mice receiving MK-28 (blue) or vehicle (red) were analysed for vocalization as number of syllables in 5 min intervals. (n = 3–4 respectively, medians +- interquartile range, Two-tailed Ttest p = 0.059).

We analysed pharmacokinetic aspects of MK-28 such as plasma stability, blood brain barrier (BBB) penetration and brain bioavailability in mice after a single IP injection of 10 mg/kg. The encouraging results showed a maximum concentration (Cmax) of 105 ng/ml and 30 min half-life in plasma, 40 min after the IP injection (Fig. 5A). In addition, very good brain bioavailability and BBB penetrance after 20 min of IP injection were also observed. The Cmax found in the brain was 57 ng/g and was obtained 40 min after the injection with a half-life of 80 minutes. Importantly, 57 ng/g is more than half of the Cmax found in plasma and the area under the curve (AUC) for the concentration in the brain was 22% of that in the plasma.

No toxic effects were observed by MK-28 administration (6 mg/kg, 6 times the dose used later for the therapeutic study) over 2 weeks of continuous delivery using subcutaneous Alzet pumps in B6 wild type mice. For therapeutic efficacy evaluation, the R6/2 HD mouse model with 160 CAG repeats was used, and subcutaneous Alzet pumps with 28-day compound delivery were placed in four-week-old mice (Fig. 5B). Given the pharmacokinetic properties of MK-28, we chose a dose of 1 mg/kg, which is well in the usual therapeutic range. Five groups were studied: 1) TG treated with MK-28 (1 mg/kg), 2) TG without treatment (pumps with vehicle), 3) Wild type treated with MK-28 (1 mg/kg), 4) Wild type without treatment (pumps with vehicle), 5) Wild type mice without pumps.

Weight evaluation during 7 weeks after pump implantation showed no significant differences between animals that received MK-28 or vehicle (p = 0.3292) (Fig. 4C). As anticipated, R6/2 mice exhibited a marked decrease in their weight in comparison to WT animals starting at week 5 after pump implantation (23.72 ± 0.59 g vs 23.99 ± 0.86 g, respectively) and until the end of the experiment by week 9 (16.34 ± 0.84 g vs 28.28 ± 1.23 g, respectively) (p < 0.001).

We assessed the motor behavior of the mice using rotarod analysis, at different time points within seven weeks, starting one week after the first MK-28 delivery. No significant differences were observed between the five groups during the first three weeks of treatment (p>0.05, Fig. 5D). Starting from the fourth week of treatment, at an age of eight weeks, significant differences in motor performance between transgenic (TG) and their control WT littermates was evident. Moreover, at this point MK-28 treated TG mice showed significant improvements in motor performance than those without treatment (p = 0.030, week 4). This effect was sustained for three weeks (p = 0.042 and p = 0.012, weeks 5 and 6, respectively) until both TG groups showed similar performances (p = 0.089) at week 7 after pump implantation (3 weeks after the end of the treatment). WT mice without any intervention performed similarly to those that received pumps filled with vehicle or MK-28 compound, showing no differences between groups and no compromised motor performance associated with MK-28 administration in WT animals.

Disease severity was evaluated using a composite phenotype test that includes the combined analysis of hind limb clasping, ledge walk, gait and kyphosis^23^. Our analysis revealed that TG and WT mice performed differently starting from week four after pump implantation (2.00 ± 1.08 vs 0.65 ± 0.79, respectively p = 0.0004, Fig. 5E), also in concordance with the rotarod results (Fig. 5D). From week 6 and until the end of the analysis (4 weeks after end of treatment), TG mice treated with MK-28 showed lower scores than those without treatment (3.14 ± 1.41 vs 4.33 ± 1.23, p = 0.016 for week 6 and 4.4 ± 0.55 vs 5.4 ± 0.89, p = 0.032 for week 8, respectively), indicating a positive effect of the treatment in gait, clasping and general motor function (Fig. 5E). Again, WT animals with or without pump or MK-28 performed similarly. As a reflection of both motor and cognitive abilities during the symptomatic phase, we measured mice vocalizations which are also affected in HD patients. The number of vocalizations were assessed in male mice when exposed to a new female in the same cage. The TG R6/2 mice produced on average 5 syllables in 5 min, while MK-28-treated mice exhibited a three-fold increase with 16 syllables in 5 min (p = 0.059, Fig. 5F). Although these results did not reach statistical significance, they show an interesting behavioral trend of improvement.

**Figure 6:**
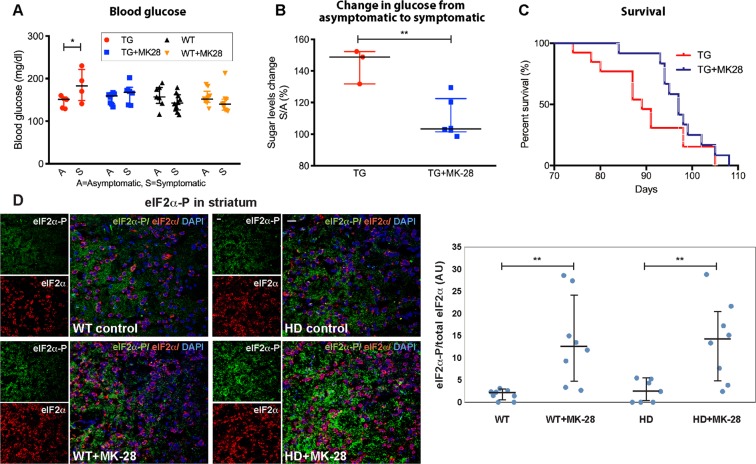
MK-28 improves systemic function and survival in R6/2 mice and induces increased levels of eIF2α-P in the mouse brain striatum. (A) Blood glucose analysis during the symptomatic phases show elevated glucose levels when compared with the asymptomatic phase for TG animals (n = 6 TG, 8 TG + MK28, 10 WT). The bars in the graph indicate medians and interquartile range. Significance * p = 0.028 Anova Newman-Keuls post hoc. (**B)** MK-28 reduced the relative increase of blood glucose between the asymptomatic and symptomatic phases. Medians and interquartile range; significance **p = 0.0045 by Two-tailed Ttest. (**C)** Survival of R6/2 mice is significantly increased upon treatment with MK-28. TG mice receiving MK-28 (blue) or vehicle (red) were analysed (n = 13/group), showing a significant increase in the survival of MK-28 treated mice (median survival 97 and 89 days, respectively, p = 0.0469). Ratio 1.887, 95% CI of ratio 0.7726 to 4.611 (Gehan-Breslow-Wilcoxon test for comparisons of Kaplan-Meier survival curves). **(D)** Analysis of the striatum in mice brain slices by immunofluorescence. eIF2α-P levels (red: total eIF2α, green: eIF2α-P) were slightly increased in the brain of the TG animals when compared to the WT at baseline, and they were significantly increased upon treatment with MK-28 in both TG and WT mice. Bar = 20 μm. The bars in the graph indicate medians and interquartile range. Significance **p (eIF2α-P WT vs. WT + MK-28) 0.003, p (eIF2α-P HD vs. HD + MK-28) 0.007, Two-tailed Ttest.

As a systemic parameter that is affected in HD patients and in some animal models during advanced stages, blood sugar levels were analysed. We found that during the asymptomatic phase no difference in blood sugar levels was observed between the TG and WT groups (144.80 ± 6.23 g/dl vs 159.14 ± 9.48 g/dl, respectively) (Fig. 6A). However, during the symptomatic phase TG mice blood sugar levels significantly increased compared to those of the asymptomatic phase (p = 0.028). In contrast, TG mice treated with MK-28 or WT control groups showed no significant increase compared to their asymptomatic levels. Analysis of the glucose increase ratio between disease stages confirmed a different behavior elicited by MK-28 treatment (p = 0.0045), where TG animals showed an increase, 144.30 ± 6.35% and TG + MK-28 maintained glucose levels similar to those of the presymptomatic phase and to their WT littermates, 109.60 ± 5.04% (Fig. 6B).

Lifespan evaluation showed that MK-28 treatment increased the median survival of TG mice by 8 days compared to controls (97d vs 89d respectively, Chi square p = 0.046; Gehan Breslow Wilcoxon p = 0.012) (Fig. 6C). In addition, death initiation was also delayed by 10 days. This observed effect on increasing survival and delaying death initiation occurred several weeks after the treatment ended.

Finally, results from the analysis of slices of mice brain striatum, at week 4 after pump implantation, show that eIF2α-P levels were slightly increased in the striatum of the TG animals when compared to the WT at baseline, but they were significantly increased upon treatment with MK-28, both for WT (~7-fold, p = 0.003) and for TG (~6-fold, p = 0.007), consistent with MK-28 reaching the brain striatum and having an activating effect on eIF2α phosphorylation (Fig. 6D).

## Discussion

ER stress is a common feature shared by diverse neurodegenerative diseases. Stress induced by accumulation of misfolded mHtt in the ER is observed in HD^7,9^. To cope with ER stress, cells activate the UPR to recover protein homeostasis or trigger apoptosis when adaptive measures are insufficient^18^. Despite our growing understanding about how the UPR alternates between those options, knowledge is still limited about their fine tuning and how to use those mechanisms for therapeutic interventions. Opposite strategies modulating ER stress through PERK pathway inhibition or activation have been tested positive in ameliorating the disease course of protein aggregation diseases^19,24–26^.

We had previously found that striatal neurons have an unusually low level of eIF2α-P, and pathogenic mHtt expression causes a strong increase in eIF2α phosphorylation by PERK^13^. Therefore, we speculated at first that inhibition of PERK could be a way to restore homeostasis and achieve therapeutic results. A4, one of the top hits in an unbiased screen for PERK inhibitors^21^, had a protective effect in cells subjected to mHtt-induced ER stress^13^. We then synthesized 5 different but related small molecules derived from A4 (Fig. 1A,B). The fact that we found that our lead compound, MK-28, is a PERK activator was thus a real surprise. MK-28 was selected by being the most efficient in rescuing ST*Hdh*^Q111/111^ cells from ER stress-induced apoptosis (Fig. 1C). MK-28 activation of PERK using purified components (Fig. 3) suggests that also its activity in cells is directly on PERK and not by an upstream event such as causing ER stress. Reevaluation of the mother compound A4 showed that it is also a PERK activator although mild (Supplementary Fig. S3). Both MK-28 and A4 were  predicted to interact with the activation loop of PERK (Fig. 4A,B). This is an interesting finding that should be verified experimentally in future studies. The activation of the eIF 2α kinase GCN2 (EIF2AK4) by MK-28. although at one order of magnitude higher than the one observed with PERK (Fig. 3B and Supplementary Fig. S2), could also be beneficial. GCN2 is also involved in the integrated stress response and its activation under stress conditions exerts cell protective effects, for example in neurodegeneration caused by ribosome stalling^27–29^. The specificity of MK-28 was further demonstrated by a highly selective activation of PERK from a panel of 391 kinases (Fig. 3C and Supplementary Table S1) and especially important is the lack of protective effect in PERK−/− cells (Fig. 4D), showing that cell rescue from ER stress-induced apoptosis by MK-28 is exclusively through PERK.

EIF2α-P levels showed a consistent but small increase in both ST*Hdh*^Q7/7^ and ST*Hdh*^Q111/111^ cells upon MK-28 treatment, coherent with a PERK activation paradigm (Fig. 2A,B). We observed a much stronger effect of MK-28 in the brains of treated R6/2 and WT mice. eIF2α-P levels were slightly increased in the striatum (the most sensitive region in HD) of R6/2 mice at baseline, similar to what we had seen before in another HD model, (N171–82Q mice)^13^. The significant further increase of eIF2α-P levels caused by MK-28 treatment (Fig. 6D) is consistent with an activating effect on eIF2α phosphorylation *in vivo*. The changes are in line with our results in ST*Hdh*^*Q7/7*^ and ST*Hdh*^*Q111/111*^ cells (Fig. 2), except that the increase in eIF2α-P levels was much more prominent in the mice. This is possibly due to the long-term effect of MK-28 in the mice, compared to the short-term treatment of the cells in culture. There might be a contribution of GCN2 activation to the eIF2α-P levels in the mice, although, as mentioned above, the results with PERK−/− cells suggest that the protective effects are exclusively through PERK. The detailed effects of MK-28 on the PERK pathway *in vivo* should be further explored in future studies. In the cultured cells it is also possible that the induction of GADD34 (Fig. 2) reduces eIF2α phosphorylation at the measured time of MK-28 treatment. Besides GADD34, we also observed upregulation of ATF4 and CHOP. The induction of CHOP by MK-28 appears to be still in the protective phase of the UPR, as it did not lead to apoptosis, but the contrary, cells were protected from ER stress. MK-28 exerts its cell-protective effect likely through the activation of the PERK pathway downstream of eIF2α-P, and not through NRF2 activation, as NRF2 inhibition did not affect cell rescue by MK-28 (Suppl. Fig. S5).

MK-28 exhibited remarkable pharmacokinetic properties in mice. It becomes readily available and is stable in plasma, penetrates the blood brain barrier and is stable inside the brain following a single IP injection (Fig. 5A). Brain levels of MK-28 were more than half of those detected in plasma, evidencing efficient bioavailability for brain tissue. We evaluated the effects of MK-28 in the HD R6/2 mouse model with 160 CAG repeats^3^, which exhibits a rapid progressive HD-like behavioral and neuropathological phenotype. This strain is recommended to be used as a gold standard for therapeutic trials in HD ^30,31^, but given the massive and very rapid progression of the disease in this model with 160 CAG repeats, sometimes the therapeutic rescue window might be too short to elicit a significant change. R6/2 mice present dystonia with limb clasping by 6 weeks, body weight loss by 5–9 weeks, reduced hindlimb and forelimb performance by 9 and 11 weeks, rotarod changes by 5 weeks, seizures and diabetes^30^. Our results were consistent with these and remarkably, our treatment ameliorated all of them while also extending mice survival (Figs. 5, 6). MK-28 was delivered continuously in 4-week-old mice for 28 days by subcutaneous minipumps, using a concentration in the range of those used in clinical settings. MK-28 exhibited long lasting effects since it lasted for several weeks after the end of the treatment (Figs. 5, 6). MK-28 treated mice showed improvements in motor performance, effect most likely induced by delayed neuronal degeneration. We were able to quantify the temporal progression of the treatment by using a composite phenotype^23^, and showed that MK-28 effectively delayed the appearance of the symptoms (Fig. 5E). Furthermore, our 28-day treatment in young mice extended median survival and death initiation by 10% (Fig. 6C). Although the motor improvement and extension of survival exhibited by MK-28 delivery in our settings might not seem dramatic, they are consistent across different evaluations of neuromotor and systemic parameters. We believe that the combination of the rapid progressing model R6/2 160 CAG, a short therapeutic intervention window and the relatively short treatment, resulted in an apparently modest improvement. We envision that our therapeutic approach could perform even better in future studies in slow progressing models with a chronic supply of the compound, which could be closer to the human disease scenario. Speech is another function generally affected in HD, since choreiform movements and degeneration of the caudate nucleus, putamen and globus pallidus also disrupt systems involved in verbal communication ^32,33^. While no signs are observed early, dysarthric speech evolve with the disease and sometimes a total communication impairment is observed^33^. In the seminal study by Mangiarini, the creators of the R6 strain, they describe chorea in face, jaw, and pharyngeal muscles affecting speech^3^. R6/1 mice emit short duration or nearly no vocalizations when compared to WT littermates^34^. We also found preliminary evidence, that R6/2 mice barely produced syllables if at all, while MK-28-treated mice exhibited a three-fold increase in the number of syllables produced within 5 min (Fig. 5F). The results are not statistically significant (p = 0.059), but show an interesting behavior of ameliorating the evaluated phenotype. We interpret this data as a trend and we treat it as merely preliminary, given the low number of animals tested. The number of animals tested was low because of the rapid death of R6/2 model mice and the late endpoint chosen for the analysis, resulting in being significantly underpowered from a statistical point of view. Future studies should include an increased number of animals to test and choose earlier endpoints, where the proportion of live animals is significantly higher. We speculate that vocalization improvements are a result of delaying neuronal malfunction and degeneration at different levels, likely cortical and striatal neurons, but we cannot pinpoint from our data whether the effects are on specific neuronal populations.

HD neuropathology is also accompanied by mHtt expression and aggregation in tissues other than the central nervous system^35^. HD subjects show diverse endocrine changes, abnormalities of carbohydrate metabolism and increased prevalence of diabetes ^36–38^. R6/2 mice exhibit pancreatic inclusions associated with hyperglycemia and insulin-deficient diabetes ^39,40^. We also found that R6/2 blood sugar levels were elevated during the symptomatic phase, but not before (Fig. 6A,B). MK-28 reduced these elevated levels to those found in WT and asymptomatic R6/2 mice. Although the number of animals that were present at both timepoints, asymptomatic (n = 3) and symptomatic stages (n = 6), was low to draw strong conclusions, the treated group behaved clearly differently from the untreated group and the difference reached statistical significance (p = 0.0045). This effect might occur through multiple mechanisms involving PERK activation.

**Figure 7:**
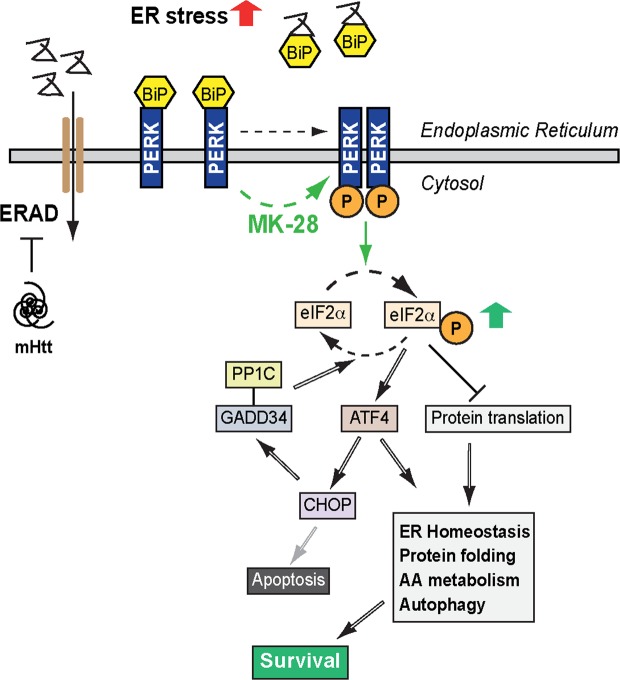
Model of the effects of mutant Htt and the action of MK-28. Accumulation of misfolded mHtt inhibits ERAD, causing ER stress and inducing the activation of the UPR to either reestablish cellular homeostasis or trigger cell death. In the activation of the PERK pathway of the UPR, unfolded ER proteins bind to the chaperone BiP, allowing oligomerization and autophosphorylation of PERK, which phosphorylates eIF2α. EIF2α-P causes a transient global suppression of protein translation, reducing the ER protein load and ER stress. EIF2α-P also promotes the translation of ATF4, upregulating beneficial UPR target genes required for autophagy, antioxidant response, and amino acid metabolism. MK-28 activates PERK, extending the beneficial transient inhibition of protein synthesis and alleviating the ER load, promoting cell survival. This strategy does not affect the activation of GADD34, which promotes the restoration of translation in the long term by reduction of eIF2α-P. This, together with the reduction in ER stress prevents initiation of apoptosis.

Altogether our results suggest that MK-28-dependent PERK activation is an effective and selective strategy to reduce ER stress, reestablish ER homeostasis and increase the survival of cells and mice expressing mHtt (Fig. 7). Consistent with this, Bruch *et al*. recently observed that PERK activation using CCT020312 is neuroprotective in a model of tauopathies with high ER stress^19^. We compared CCT020312 to MK-28, to find that MK-28 was more efficient (with an IC50 ~3-fold higher) in rescuing mHtt-expressing striatal neurons from apoptosis (Fig. 4C). This mechanism is consistent with previous reports using another strategy for PERK pathway activation in other protein aggregation disorders, by inhibiting eIF2α-P dephosphorylation ^26,41–43^. This is accomplished by inhibition of the eIF2α-P phosphatase regulatory subunit GADD34 (PPP1R15A) or the constitutive subunit CREP (PPP1R15B). GADD34 is induced downstream of ATF4, resulting in recovery from the PERK-mediated block in protein synthesis^44^. This strategy was also beneficial in a cellular model of HD (PC6.3 cells expressing mHtt fragments)^10^ and in an HD mouse model (N171–82Q)^43^. This approach may prolong the benefit of transient inhibition of protein synthesis by eIF2α-P, but has the caveat that restoration of protein synthesis in the long term by eIF2α-P dephosphorylation is also inhibited, and in turn, pro-apoptotic pathways may be activated. In fact, GADD34 inhibition had negative effects in at least one report, where a mutant SOD1 ALS mouse model was used^45^. In contrast, our approach of directly activating PERK stimulates the beneficial transient inhibition of protein synthesis, alleviating the ER load, and does not affect the activity of CREP or the activation of GADD34, consequently avoiding pro-apoptotic outcomes and restoring translation in the long run (Fig. 7).

The benefit of PERK activation is in line with the fact that loss of functional PERK (Wolcott-Rallison syndrome) or PERK missense mutations cause neurodegeneration ^46,47^. Although PERK inhibition showed beneficial effects for some diseases ^24,25,48–52^, the side effects of chronic treatment are of major importance. A strong PERK inhibition as that caused by GSK2606414, can lead to a Wolcott-Rallison syndrome-like phenotype produced by the loss of function of PERK^53^. The benefit of transient inhibition of protein synthesis by eIF2α-P is not achieved, and this is detrimental and toxic to neurons as well as secretory tissues, such as the pancreas.

The compound that we developed, MK-28, was not toxic at high concentrations, although this was tested only in the short-term and should be analysed in the long term for the possibility of deleterious effects. MK-28 exhibited a notable pharmacokinetic profile and brain bioavailability in mice. Moreover, our compound showed a selective and efficient PERK activation effect that was sufficient to rescue cellular and animal models of HD. Our data support further evaluation of MK-28 in slow progressing HD models such as BACHD and knock-in models, since those more closely resemble the slow pathological progression observed in humans. Currently, there is a complete lack of selective therapeutic options for HD, thus we consider MK-28 as a potential safe therapeutic agent for this disease. Finally, since ER stress is also present and drives all of the most prevalent protein misfolding diseases, we speculate that MK-28 could also be effective in those related pathological scenarios.

## Methods

### Study design

The objective of this study was to evaluate preclinically the safety, kinetics and efficacy of a new compound that we synthesized, a derivative of a PERK modulator, in models of HD. We determined that the compound is a selective PERK activator. A therapeutic strategy based on the over-activation of PERK represents an unexplored and promising approach for treating HD, where protein aggregation drives the pathological progression of the disease and the development of ER stress is a main cytotoxic factor. We first investigated the effects of several small molecule derivatives of the PERK modulator A4 in a cellular model of HD and also under acute ER-stress. For all the *in vitro* studies, at least three independent experiments with internal replicates were performed. We then selected a compound (MK-28) with the best cytoprotective effects on a neuronal model of HD. This compound, which activated PERK with purified components *in vitro*, was further tested *in vivo* in a mouse HD model, the R6/2 strain harboring 160 CAG repeats. Power calculations determined that 16 mice per group was the minimum required to see at least a 20% increase (alpha 0.05, beta 0.2, power 80%) in motor performance and survival of R6/2 mice with MK-28 subcutaneous delivery compared to vehicle. Mice were coded and randomly assigned to different groups. Investigators were blinded to the different treatment groups during the experiment. Multiple endpoints were prospectively selected for the *in vivo* part, consistent with the analyses we here report. Data collection was ended before signs of animal suffering or inability to thrive appear. All *in vivo* experiments followed institutional guidelines and were approved by the Institutional Animal Care and Use Committee of Tel Aviv University.

### Materials

Tun, thapsigargin, ML385 and common reagents were from Sigma. GSK606414 was from APExBio and CCT020312 from Calbiochem. Mouse anti-actin and rabbit anti-calnexin antibodies were from Sigma. Mouse anti-eIF2α was from Cell Signaling. Rabbit anti-phospho-eIF2α (Ser51) was from MBL. Rabbit anti-PERK was from Abcam. Rabbit anti-ATF4 was a kind gift from David Ron (Univ. Cambridge). Goat anti-mouse IgG conjugated to 488 DyeLight, goat anti-rabbit IgG conjugated to 564 DyeLight, and goat anti-rabbit and anti-mouse IgG conjugated to HRP were from Jackson Labs. Normal goat serum was from Vector Laboratories (Burlingame, CA).

### Compound generation

See detailed information in Supplementary methods.

### Toxicity assay in cells in culture

ST*Hdh*^Q111/111^ cells (used before^13^) or PERK+/+ or PERK−/− MEFs (kindly provided by David Ron, Univ. Cambridge) were incubated with Tun (10 μg/ml) for 48 h, which results in ER stress-induced cell death. Cells under the same conditions were incubated for the same times, together with Tun, with increasing concentrations of the different PERK modulators (0–120 μM) and the percent of apoptotic cells was measured by FACS cell cycle progression using propidium iodide (PI), considering the fraction beneath G0/G1. The relative apoptosis rate was calculated to be able to compare between compounds and experiments, where for each concentration curve the percent of apoptotic cells was considered 100 at concentration 0 of the PERK modulator, with only Tun. The graphs show the average relative apoptosis rate of at least 3 independent experiments for each compound.

### Cell Cycle FACS analysis

Cells were washed with PBS and fixed with cold methanol. Before FACS analysis cells were washed with PBS, followed by addition of propidium iodide (PI) solution (10 μg/ml) and analysis by flow cytometry. Cells in sub-G0/G1 were counted as apoptotic/ dead.

### Treatments and immunoblotting

ST*Hdh*^*Q7/7*^ and ST*Hdh*^*Q111/111*^ cells were treated with different concentrations of MK-28. The concentration range was chosen in accordance to previous studies with A4. Cells were lysed with PBS containing 1% Triton X-100, 0.5% Sodium deoxycholate with protease and phosphatase inhibitors cocktails. Cocktail 2 and 3 (Sigma) were added to the lysis buffer for detection of phosphorylated proteins and lysates were incubated on ice for 30 minutes, then centrifuged at 16,000xg for 30 minutes a 4 °C. The supernatants were boiled in loading buffer for 5 min and loaded on 12% reducing SDS-PAGE, except for PERK where 6 M urea was added before boiling and 8% SDS-PAGE was used. Immunoblotting, detection by ECL and quantitation was done as described previously^13^.

### Real time PCR

Total cell RNA was extracted with TRIzol reagent (Invitrogen) according to the manufacturer's instructions. Reverse transcription (RT) was performed with QuantaBio qScript cDNA synthesis kit. Real-time qPCR was carried out using a CFX Connect Real-Time PCR Detection System (BioRad) and the amplifications were done using the SYBR Green PCR Master Mix (pcrbio). Reactions were run in triplicates in three independent experiments. Housekeeping gene GAPDH was used as an internal control to normalize the variability of expression levels.

### *In vitro* hotspot kinase assay

*In vitro* kinase assays with purified components and full kinase panel analysis was done at Reaction Biology Corp. (Malvern, PA, USA) using the HotSpot Kinase Assay. Reactions were performed for 20 min at room temp. using ^33^P-ATP (Specific activity 10 μCi/μl) with 1 μM total ATP, 20 μM substrate and kinases in the nanomolar range in 20 mM Hepes (pH 7.5), 10 mM MgCl2, 1 mM EGTA, 0.02% Brij35, 0.02 mg/ml BSA, 0.1 mM Na3VO4, 2 mM DTT, 1% DMSO and other required factors depending on the assayed kinase. GST-tagged, recombinant human, catalytic domain (amino acids 536–1116) of PERK, expressed in insect cells was used (Life technologies, Carlsbad, CA).

### Pharmacokinetics and BBB penetration

See detailed information in Supplementary methods.

### Huntington's disease mouse model

All experimental procedures were approved by and conducted in strict compliance with the Institutional Animal Care and Use Committee of Tel Aviv University. Mice were kept in a 12/12-hour light/dark cycle with food and water ad libitum. The HD mouse model used was the R6/2 TG mice model B6CBA-R6/2 (CAG 160) (Jackson Lab, catalog number #002810, USA). At three weeks mice were assessed for CAG repeat size using the following primer set: 5’-ATGAAGGCCTTCGAGTCCCTCAAGTCCTTC-3′ and 5′-GGCGGCTGAGGAAGCTGAGGA G-3′. Following Jackson laboratories guidelines, wild type (WT) and TG littermates were kept combined in the same minimally-enriched cages (with cardboard rolls and gauze pads), and after week 8 water and mushed food were placed at the ground level for all the cages. Male mice treatment groups were coded and remained blind to the observers throughout the experiment. Power calculations determined that 16 mice per group was the minimum required to see at least a 20% increase (alpha 0.05, beta 0.2, power 80%) in motor performance and survival of R6/2 mice with MK-28 subcutaneous delivery compared to vehicle. Multiple endpoints were prospectively selected, consistent with the analyses we here report. Data collection was ended before signs of animal suffering or inability to thrive appear.

### Osmotic minipump preparation and implantation

Alzet pumps model 1004 (Alzet, USA), with a rate of 0.11 µl/hour and 28 days effective compound delivery was used. MK-28 compound was dissolved in 1:1 DMSO/PEG400 and prepared to delivery subcutaneously 6 mg/kg MK-28 for toxicity studies (n = 8) or 1 mg/kg for therapeutic evaluation in R6/2 mice (n = 72). For toxicity studies mice receiving six times the therapeutic dose of MK-28 or vehicle were weighed every two days for two consecutive weeks. For the therapeutic evaluation of MK-28, five groups of mice were studied: 1) R6/2 TG mice treated with MK-28, 2) TG without treatment (pumps with vehicle DMSO/PEG400 1:1), 3) WT treated with MK-28, 4) WT without treatment (pumps with vehicle DMSO/PEG400 1:1), 5) WT without pumps. Alzet pumps were implanted in 4 weeks old mice and MK-28 was administered at 1 mg/kg with continuous dosing for 28 days. All mice were anaesthetized with ketamine (80 mg/kg IP) and xylazine (10 mg/kg IP) before subcutaneous pump implantation.

### Rotarod test

Motor activity was assessed by the rotarod test (San Diego Instruments, CA, USA) (0–25 RPM). Relative slow acceleration was used to discriminate small differences between groups particularly at advanced stages of the disease. All mice were trained together before any experimental procedure was performed to set a common baseline. They were required to stay at least 2 minutes without falling in order to participate in the study. R6/2 mice and control littermates were tested every week after pump transplantation. Each test consisted of 3 consecutive measurements of 5 minutes with inter-trial recess of 5 minutes. The latency to fall was recorded and the best performance of the three trials was considered for the analysis.

### Combined phenotype

A rapid and sensitive combined phenotype score was used to quantify disease severity in HD mouse model^23^. Measures include hind limb clasping, ledge test, gait and kyphosis. See detailed information in Supplementary methods.

### Glucose levels

For blood glucose measurements, blood samples from all the evaluated groups (n = 34) were obtained from the tail and measured with the Accu-Check glucose monitor (Roche Diagnostics, USA). Two independent glucose measurements were performed at the same hour 3 and 12 weeks after birth corresponding to the asymptomatic and symptomatic stages respectively. Glucose levels are expressed in mg/dl.

### Ultrasonic vocalizations

Ultrasonic vocalizations were recorded on males during the first 5 minutes of female exposure, 12 weeks after birth. Vocal emissions were monitored by an UltraSoundGate Condenser Microphone (CM 16; Avisoft Bioacoustics, Berlin, Germany) placed 16 cm above the floor of each male's cage. This microphone is sensitive to frequencies of 15–180 kHz with a flat frequency response (±6 dB) between 25–140 kHz. Microphones were connected via an Avisoft UltraSoundGate 416 USB Audio device (Avisoft Bioacoustics) to a personal computer, acoustic data was displayed with an Avisoft RECORDER (version 2.97; Avisoft Bioacoustics), recording at 300,000 Hz in16 bit format. For acoustical analysis, recordings were transferred to SASLab Pro (version 4.50; Avisoft Bioacoustics) and a fast Fourier transform was conducted (512 FFT length, 100% frame, Hamming window and 75% time window overlap). Spectrograms were produced at 586 Hz of frequency resolution and 0.427 ms of time resolution. Figure plotted by MATLAB code.

### Brain immunohistochemistry

Under anesthesia, animals were perfused with 4% PFA (formaldehyde in phosphate-buffered saline (PBS)) and the brains were removed and soaked in 4% PFA overnight. Then, brains were incubated overnight in 30% sucrose solution and frozen in OCT compound Tissue-Tek by using dry ice. Sections (10 µm) of the striatum were obtained by cryostat. Brain sections were processed for immunochemistry with the kind help of Daniel Frenkel. They were washed with PBS for 10 minutes, incubated in 0.5% triton-X-100 for 10 minutes and then in blocking solution containing 1% bovine serum albumin (BSA), 10% normal goat serum and 0.25% triton for one hour at room temperature. The samples were rinsed with PBS and incubated overnight at 4 °C with the selected primary antibody diluted in blocking solution (eIF2α-mouse [1:400] and phosphorylated eIF2α-rabbit [1:400]). Samples were washed 3 times with PBS and incubated with the proper secondary antibody (564 DyeLight Anti-Rabbit [1:400] or 488 DyeLight Anti-mouse [1:400]) for 2 hours at room temperature. After washing with PBS, samples were placed in DAPI (4′,6-diamidino-2-phenylindole) diluted in PBS for 10 minutes, mounted and examined by confocal microscope Zeiss LSM-510, 3 slides were analysed from each subject.

### Image analysis

Signal quantification and cell counting were done using the ImageJ program. The value obtained for the phosphorylated eIF2α signal was divided by the total eIF2α signal in the same image. The result was then divided by the number of cells (identified by DAPI staining) for normalization. The final value for each subject was an average of images of 2–4 samples for each group. Two-tailed, paired sample student's t tests were performed. Statistical significance was determined at P < 0.05.

### Computational homology modeling

The three-dimensional structure of human PERK without the activation loop (residues 958–984) is available at the Protein Data Bank (PDB code: 4G34)^22^. The binding site of the inhibitor GSK6924 is in the vicinity of the activation loop, thus this loop was homology modeled based on the non-phosphorylated tyrosine kinase (PDB code: 1OPJ). MODELLER 9.17^54^ was used for the homology modeling of the activation loop using default parameters.

### Computational docking of compounds

Docking studies were performed using Glide SP (standard precision)^55^ as implemented in Maestro 11.3. Prior to docking the protein structure was prepared (i.e. missing hydrogen atoms were added, protonation states of titratable residues were determined, and restrained energy minimization of hydrogen atoms was performed to remove clashes) using the protein preparation^56^ wizard in Maestro 11.3. The compounds (A4, MK26, MK28, MK29, and MK30) were 3D modeled using LigPrep, which produces chemically correct structures, and then were subjected to a conformational search protocol in Maestro 11.3. Representative conformations of the ligands were used as input for docking calculations. For each ligand, poses were selected based on Glide Emodel Score energies, and analysed for their interactions with binding site residues. The ranking of the interactions of the different analogues in the binding site was based on the Glide Score (gscore).

### Statistical analysis

The results are expressed as mean ± standard error unless otherwise stated. Student's t-test (two-tailed) was used to compare the means of two groups. Comparisons between several groups were performed using ANOVA with Scheffe posthoc analysis. Repeated tests (weekly rotarod) were also analysed using the Two-Way ANOVA test. Statistical significance was determined at P < 0.05 (*), P < 0.01 (**), P < 0.001 (***).

### Ethics approval

All *in vivo* experiments followed institutional guidelines and were approved by the Institutional Animal Care and Use Committee of Tel Aviv University.

## Supplementary information

Supplementary information.

Supplementary Table S1.

## Data Availability

All data generated or analysed during this study are included in this published article and its supplementary information files.
