# Supplementary material for: A novel specific PERK activator reduces toxicity and extends survival in Huntington's disease models
Source: Sci Rep. 2020 Apr 23;10:6875. doi: 10.1038/s41598-020-63899-4 (PMC7181660; doi:10.1038/s41598-020-63899-4)
Supplement: Supplementary file 1 — Supplementary information. [file 41598_2020_63899_MOESM1_ESM.pdf]

# A novel specific PERK activator reduces toxicity and extends survival in Huntington’s disease models

Javier Ganz, Talya Shacham, Maria Kramer, Marina Shenkman, Hagit Eiger, Nitai Weinberg, Ori Iancovici, Somnath Roy, Luba Simhaev, Benny Da’adoosh, Hamutal Engel, Nisim Perets, Yael Barhum, Moshe Portnoy, Daniel Offen and Gerardo Z. Lederkremer

## Supplementary information

## Supplementary methods

### Compound generation - Chemical Synthesis

General procedure for the preparation of 2-[N-Methyl-N'-(benzylidene)hydrazinyl]-4,6-diphenylpyrimidines.

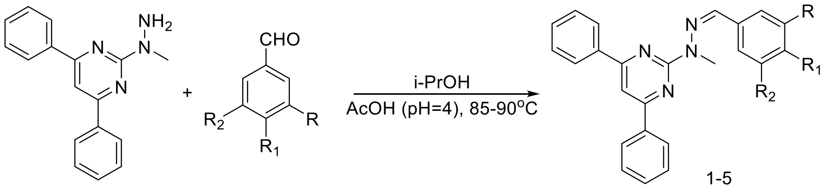


To a suspension of 2-(N-methylhydrazino)-4,6-diphenylpyrimidine (1eq) in isopropyl alcohol (7-8 mL) was added the corresponding substituted benzaldehyde (2eq). Acetic acid was then added until pH 4 was reached, and the reaction mixture was refluxed for 2 h, until TLC (DCM) has indicated disappearance of the starting materials and the formation of the product. After cooling the reaction mixture in refrigerator overnight, the resultant yellow precipitate was filtered off, washed with cold isopropanol and dried under high vacuum.

Synthesis of 2-[N-methyl-N'-(3-methoxy-4-hydroxybenzylidene)hydrazinyl]-4,6-diphenylpyrimidine.

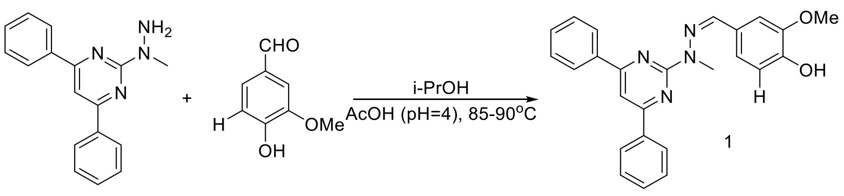


The compound 1 was synthesized by refluxing of 2-(N-methylhydrazino)-4,6-diphenylpyrimidine (0.13 g, 0.45 mmol, 1 eq) and 3-methoxy-4-hydroxybenzaldehyde (vanillin) (0.14 g, 0.9 mmol, 2 eq) in 8 mL isopropanol in the presence of the AcOH, as described by the general procedure. The yield of light yellow crystals of 1 was 0.15 g (80 %). $^{1}$H NMR (400 MHz, DMSO-d$^{6}$) δ: 9.36(br s, 1H), 8.45(d, J = 5.8 Hz, 4H), 8.10(s, 1H), 7.95(s, 1H), 7.58(m, 6H), 7.17(d, J = 8.1 Hz, 1H), 6.86(d, J = 8.0 Hz, 1H), 3.93(s, 3H), 3.79(s, 3H). $^{13}$C NMR (100 MHz, DMSO-d$^{6}$) δ: 165.7, 149.4, 148.7, 138.7, 138.1, 132.2, 129.9, 128.3, 122.5, 56.4, 32.8, 26.7.

Synthesis of 2-[N-methyl-N'-(3,5-dimetoxybenzylidene)hydrazinyl]-4,6-diphenylpyrimidine

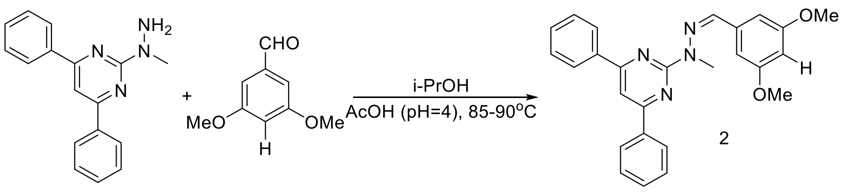


The compound 2 was synthesized from 2-(1-methylhydrazino)-4,6-diphenylpyrimidine (0.1 g, 0.36 mmol, 1 eq) and 3,5-dimethoxybenzaldehyde (0.12 g, 0.72 mmol, 2 eq) in 8 mL isopropanol in the presence of AcOH, as described above by the general procedure. The yield of white crystals of 2 was 0.13 g (86 %). $^{1}$H NMR (400 MHz, DMSO-d$^{6}$) δ: 8.45(m, 4H), 8.15(s, 1H), 7.90(s, 1H), 7.58(dd, J = 2.0 Hz , J = 5.4 Hz, 6H), 7.11(d, J = 2.2 Hz, 2H), 6.50(t, J = 2.2 Hz, J = 4.7 Hz, 1H), 3.84(s, 6H), 3.83(s, 3H). $^{13}$C NMR (100 MHz, DMSO-d$^{6}$) δ: 165.9, 161.1, 137.3, 131.5, 129.3, 127.6, 104.6, 101.6, 55.5, 31.4.

Synthesis of 2-[N-methyl-N'-(3,4-dihydroxybenzylidene)hydrazinyl]-4,6-diphenylpyrimidine.

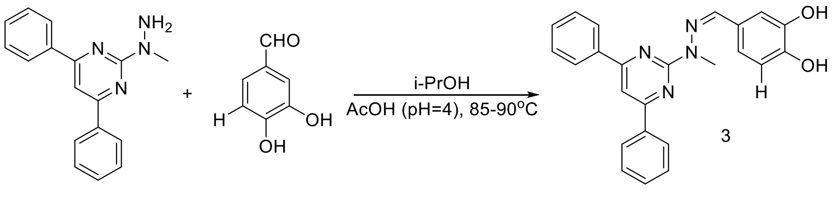


The compound 3 was synthesized from 2-(1-methylhydrazino)-4,6-diphenylpyrimidine (0.1 g, 0.36 mmol, 1 eq) and 3,4-dihydroxybenzaldehyde in 8 mL isopropanol in the presence of AcOH, according to the general procedure. The yield of 3 was 0.12 g (84 %).

$^{1}H$ NMR (400 MHz, DMSO-$d^6$) δ: 9.24(br s, 1H), 8.41(m, 4H), 8.06(s, 1H), 7.90(s, 1H), 7.60(dd, J = 1.9 Hz, J = 5.8 Hz, 6H), 7.39(d, J = 1.9 Hz, 1H), 7.07(dd, J = 1.9 Hz, J = 8.2 Hz, 1H), 6.81(d, J = 8.1 Hz, 1H), 3.79(s, 3H).

$^{13}C$ NMR (100 MHz, DMSO-$d^6$) δ: 165.9, 147.9, 146.7, 140.0, 138.2, 132.0, 128.8, 128.4, 120.9, 116.7, 114.1, 104.9, 63.2, 32.4, 26.6.

Synthesis of 2-[N-methyl-N'-(3-hydroxy-4-methoxybenzylidene)hydrazinyl]-4,6-diphenylpyrimidine.

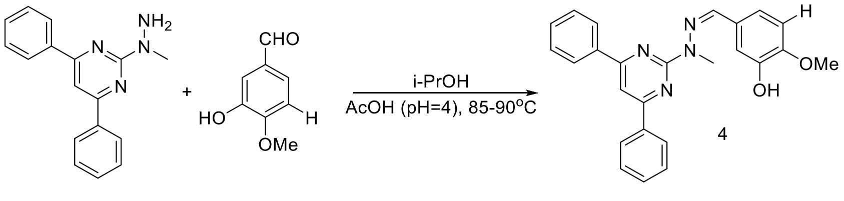


The compound 4 was synthesized from 2-(1-methylhydrazino)-4,6-diphenylpyrimidine (0.1 g, 0.36 mmol, 1 eq) and 3-hydroxy-4-methoxybenzaldehyde (0.115 g, 0.72 mmol, 2 eq) in 8mL isopropanol in the presence of AcOH, as described by the general procedure. The yield of yellow crystals of 4 was 0.1 g (68 %).

$^1H$ NMR (400MHz, DMSO-$d^6$) δ: 9.29(br s, 1H), 8.42(m, 4H), 8.08(s, 1H), 7.94(s, 1H), 7.58(m, 6H), 7.42(d, J = 5.1 Hz, 1H), 7.20(dd, J = 2.6 Hz, J = 9.1 Hz, 1H), 7.01(d ,J = 10.2 Hz, 1H), 4.39(d, J = 5.1 Hz, 1H), 3.82(d, J = 1.7 Hz, 6H).

$^{13}C$NMR (100MHz, DMSO-$d^6$) δ: 165.9, 162.0, 150.6, 147.9, 140.6, 138.1, 132.1, 130.0, 128.4, 121.2, 114.1, 114.0, 105.7, 63.9, 56.9, 32.8, 26.6.

Synthesis of 2-[N-methyl-N'-(3,5-dimethoxy-4-hydroxybenzylidene)hydrazinyl]-4,6-diphenylpyrimidine.

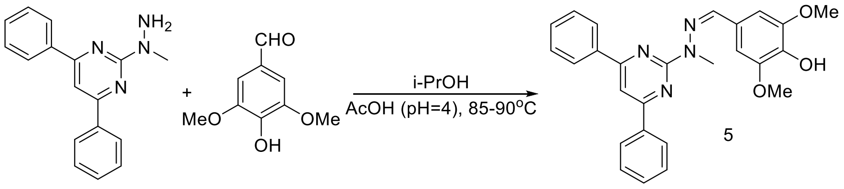


The compound 5 was synthesized from 2-(1-methylhydrazino)-4,6-diphenylpyrimidine (0.1 g, 0.36 mmol, 1 eq) and 3,5-dimethoxy-4-hydroxybenzaldehyde (0.13 g, 0.72 mmol, 2 eq) in 8 mL isopropanol in the presence of AcOH, as described by the general procedure. The yield of yellow crystals of 5 was 0.13 g (82 %).

$^1H$ NMR(400 MHz, DMSO-$d^6$) δ : 8.71(br s, 1H), 8.43(m, 4H), 8.09(s, 1H), 7.93(s, 1H), 7.56(dd, J = 1.8 Hz, J = 5.2 Hz, 6H), 7.22(s, 2H), 3.89(s, 6H), 3.81(s, 3H).

$^{13}C$ NMR (100 MHz, DMSO-$d^6$) δ: 165.8, 149.3, 138.6, 132.2, 129.9, 128.3, 113.3, 104.9, 56.8, 2.0.

**Pharmacokinetics and BBB penetration**

Pharmacokinetics evaluation was performed by Pharmaseed Ltd. (Ness Ziona, Israel). The study was performed according to group allocation and study time line on 24 female mice as indicated in the following table.

<table>
<tr><th>Group #</th><th>Animals #</th><th>Treatment</th><th>Dose Level (mg/kg)</th><th>Dose Volume (ml/kg)</th><th>Route of administration</th><th>Terminal bleeding and brain removal</th></tr>
<tr><td>1F</td><td>3</td><td>Vehicle</td><td>10</td><td>10</td><td>IP</td><td>0min</td></tr>
<tr><td rowspan="2">2F</td><td>1</td><td rowspan="9">MK-28 (Test Item)</td><td rowspan="9">10</td><td rowspan="9">10</td><td>IV</td><td rowspan="2">5min</td></tr>
<tr><td>2</td><td>IP</td></tr>
<tr><td>3F</td><td>3</td><td>IP</td><td>20min</td></tr>
<tr><td>4F</td><td>3</td><td>IP</td><td>40min</td></tr>
<tr><td>5F</td><td>3</td><td>IP</td><td>60min</td></tr>
<tr><td>6F</td><td>3</td><td>IP</td><td>2h</td></tr>
<tr><td>7F</td><td>3</td><td>IP</td><td>4h</td></tr>
<tr><td>8F</td><td>3</td><td>IP</td><td>6h</td></tr>
<tr><td>9F</td><td>3</td><td>IP</td><td>24h</td></tr>
</table>

Determination of MK-28 levels in the plasma and brain were performed by LC/MS/MS using generic methods in the bioanalytical lab of WuXi AppTec Co., Ltd. (Shanghai, China). The MK-28 concentrations were determined in the plasma and whole perfused brain tissues of the animals following a single IP injection of 10 mg/kg.

**Combined phenotype:** A method assessing a combination of different disease-phenotypes was used to evaluate disease progression [33]. Phenotypic evaluation includes hind-limb clasping, ledge test, gait and kyphosis. *Limb clasping:* hind-limb clasping behavior was evaluated and different scores were given based on their performance on a scale from 0 to 3: 0 = normal; 1 = clasps hind-limbs within 30s of being suspended in the air; 2 = clasps hind-limbs within 5s, recovering when released after 30s; 3 = clasps hind-limbs within 5s and has difficulty recovering when released after 30s. *Ledge test:* The ledge test evaluates motor coordination by

placing mice on the ledge of the cage. Typically, mice will walk along the ledge and attempt to descend back into the cage. Walking without losing balance is considered normal and assigned a score of 0. A score of 1 is given if the mouse misses its footing while walking. If hind-legs are not used, or lands on its head when descending, a score of 2 will be given. Score of 3 is assigned if it falls off the ledge, or shakes and refuses to move at all despite encouragement. *Gait:* Evaluates coordination and muscle function. A normal mouse using four limbs equally and with good weight support receives a score of 0. Limping while walking, receives a score of 1. Severe limping and lowered pelvis, receives a score of 2. If the mouse moves with difficulties dragging his body, it receives a score of 3. *Kyphosis:* Refers to the characteristic dorsal curvature of the spine manifested in animal models of neurodegenerative diseases. If the mouse keeps its spine straight as it walks, it receives a score of 0. Mild kyphosis is given a score of 1 and inability to straighten its spine completely, with persistent mild kyphosis is scored as 2. Mice exhibiting pronounced kyphosis is assigned a score of 3.

## Supplementary figures

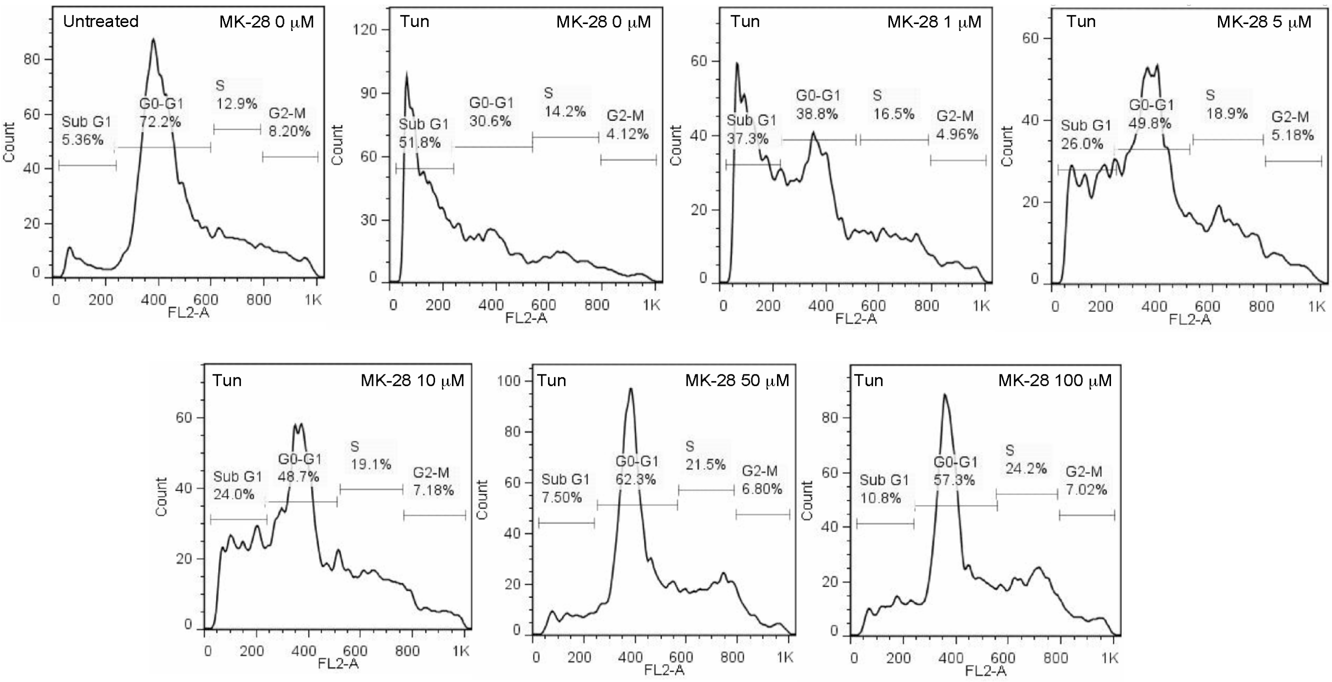


**Suppl. Fig. S1. Cell Cycle FACS analysis and the rescue effect of MK-28.** Representative graphs of apoptosis rescue analysis corresponding to the experiment in Fig. 1C, using ST*Hdh*$^{Q111/111}$ cells incubated with Tun and the indicated concentrations of MK-28. Cells were incubated with propidium iodide and analyzed by flow cytometry, the sub-G1 peak represents apoptotic/dead cells.

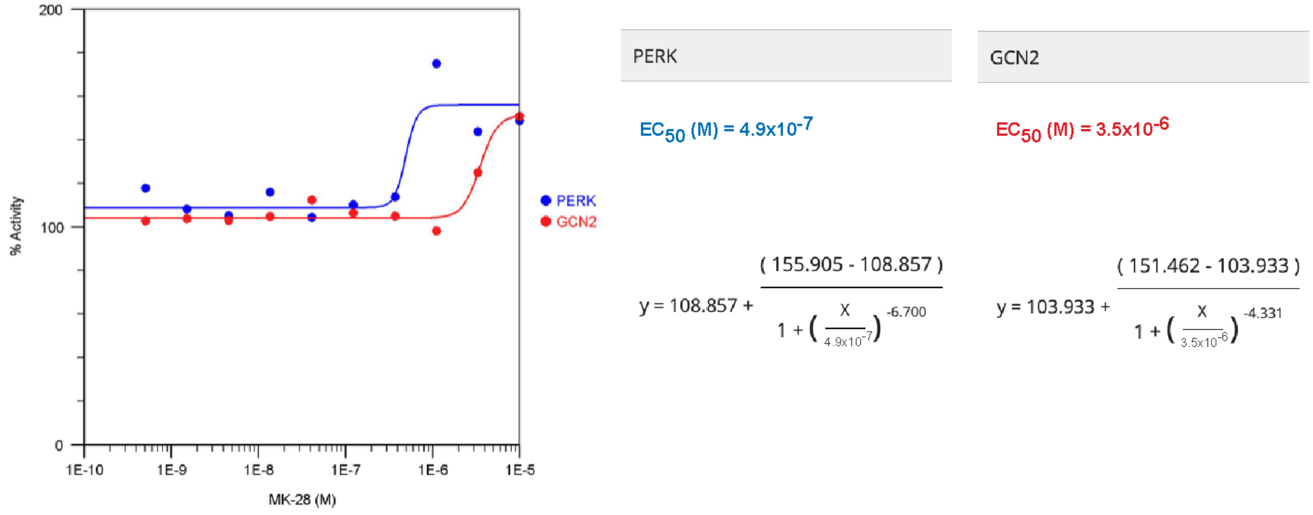


**Suppl. Fig. S2. EC50 calculation for MK-28 activation.** MK-28 EC50 for PERK and GCN2

was calculated from the data in Fig. 3. Note that for the calculation for GCN2, we considered that the plateau was reached at 10 µM, if this is not so the EC50 would be even higher.

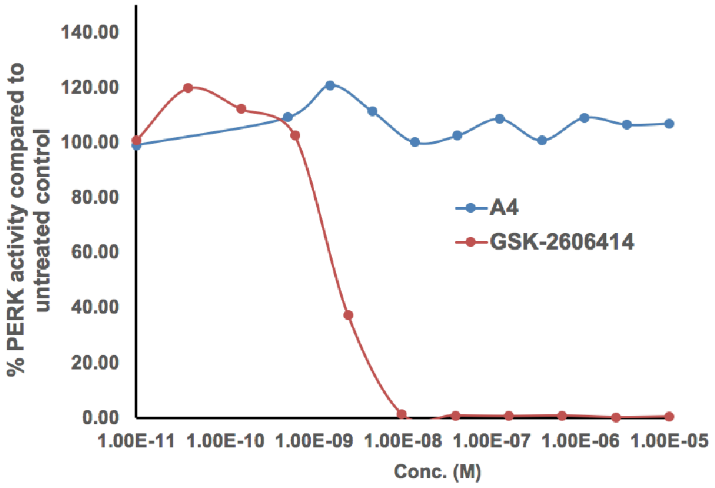


**Suppl. Fig. S3. Modulation of PERK by A4.** A4 was tested for its effect on isolated PERK *in vitro*, showing a weak activating effect in the nanomolar range. (performed at Reaction Biology Corp. using the HotSpot Kinase Assay).

**A**

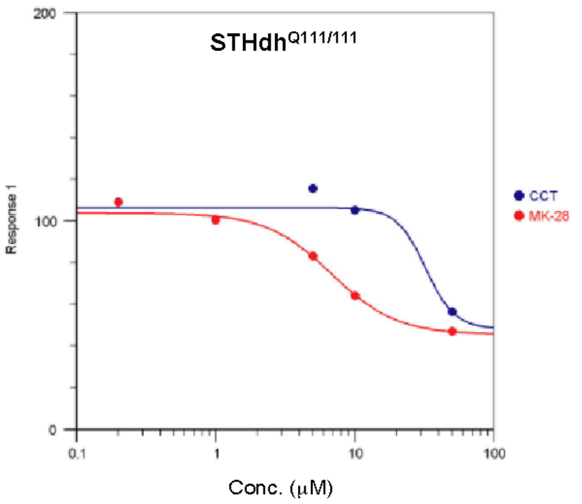


| MK-28 | CCT |
| --- | --- |
| $IC_{50} = 6.794$ | $IC_{50} = 32.389$ |
| $y = 45.396 + \frac{(103.759 - 45.396)}{1 + \left(\frac{x}{6.794}\right)^{1.857}}$ | $y = 48.167 + \frac{(106.239 - 48.167)}{1 + \left(\frac{x}{32.389}\right)^{4.159}}$ |

**B**

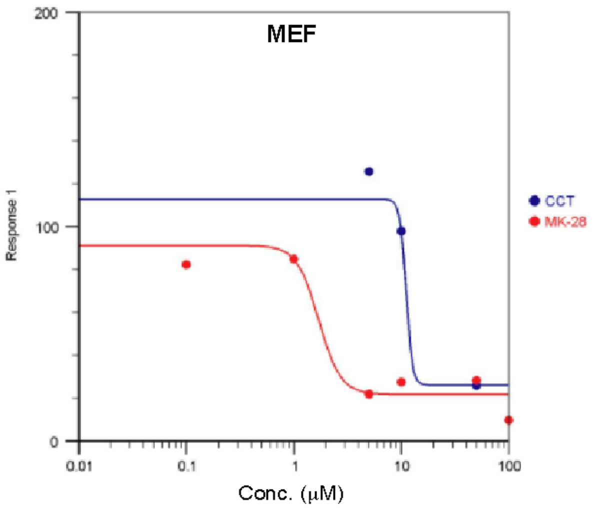


| MK-28 | CCT |
| --- | --- |
| $IC_{50} = 1.715$ | $IC_{50} = 11.116$ |
| $y = 21.794 + \frac{(91.217 - 21.794)}{1 + \left(\frac{x}{1.715}\right)^{4.273}}$ | $y = 25.972 + \frac{(112.865 - 25.972)}{1 + \left(\frac{x}{11.116}\right)^{14.870}}$ |

**Suppl. Fig. S4. IC50 calculation for inhibition of apoptosis.** IC50 for the protection from apoptosis by MK-28 compared to CCT020312 in ST*Hdh*$^{Q111/111}$ cells (**A**, from data in Fig. 3C) or in WT MEF cells (**B**, from data in Fig. 3D).

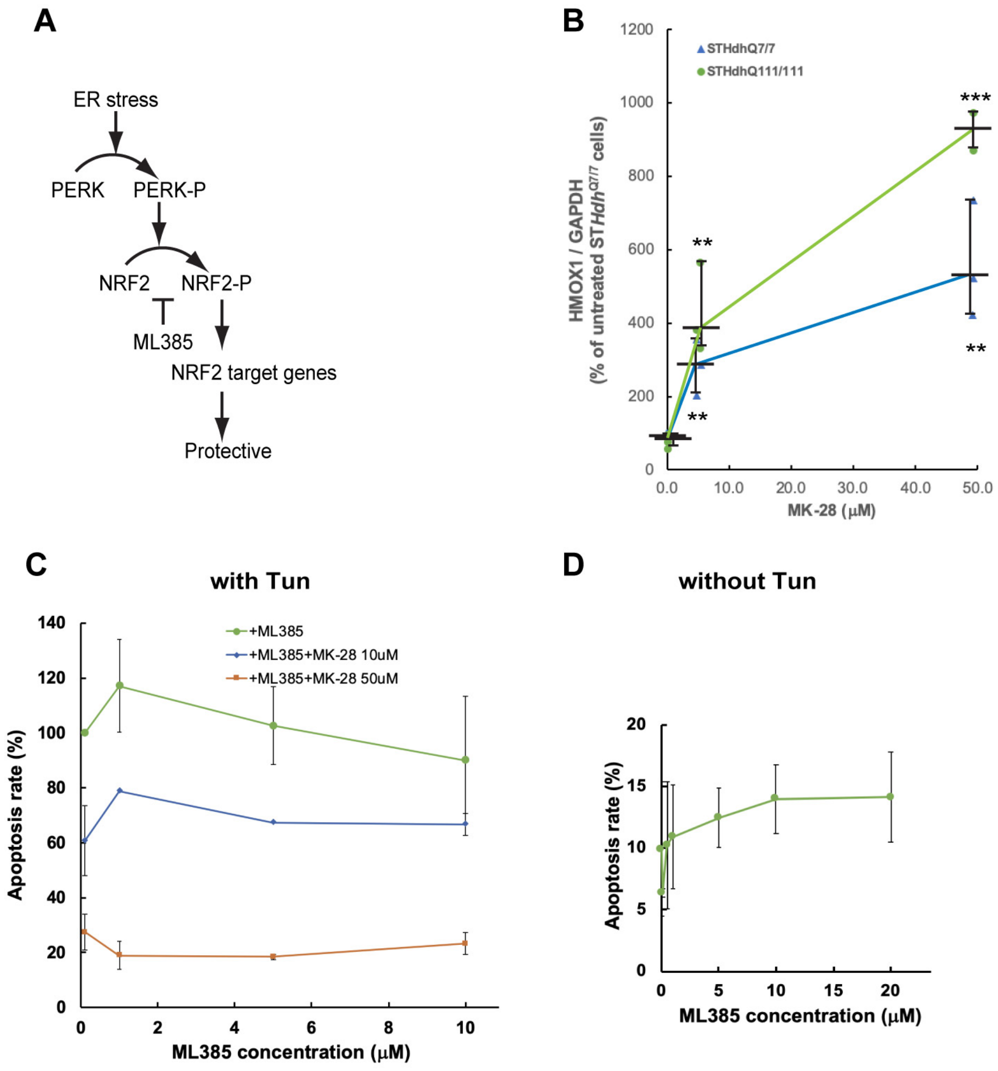


**Suppl. Fig. S5. NRF2 activation but not involvement in cell protection by MK-28**. **A)** Scheme of NRF2 activation by PERK. **B)** qPCR of HMOX1, a downstream target of NRF2. Medians +- interquartile range of 3 independent experiments. P values of ST*Hdh*$^{Q7/7}$ MK 5µM vs. untr = 0.01, ST*Hdh*$^{Q7/7}$ MK 50µM vs. untr = 0.007, ST*Hdh*$^{Q111/111}$ MK 5µM vs. untr = 0.008, ST*Hdh*$^{Q111/111}$ MK 50µM vs. untr =0.0002. **C)** Apoptosis of ST*Hdh*$^{Q111/111}$ cells in the presence of Tun and the indicated concentrations of MK-28, plus increasing concentrations of the NRF2 inhibitor ML385. Average of 3 independent experiments +-SD. **D)** Apoptosis of ST*Hdh*$^{Q111/111}$ cells treated only with increasing concentrations of ML385. Average of 3 independent experiments +-SD.

Fig. 2B:

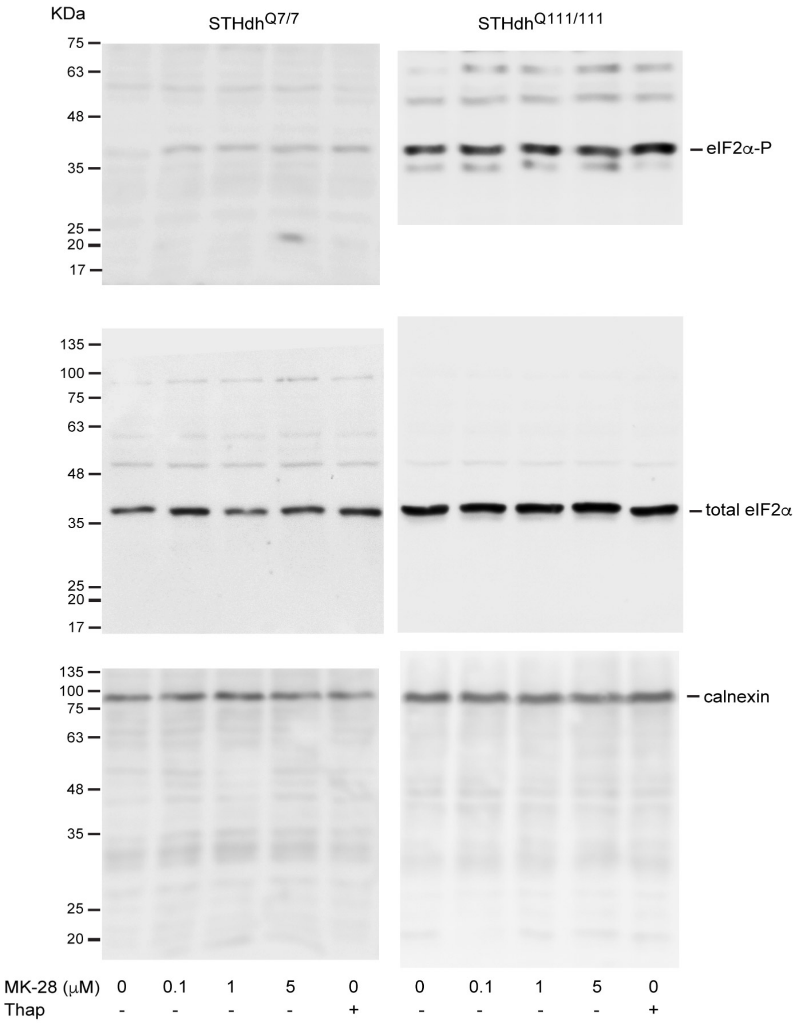


Fig. 2C:

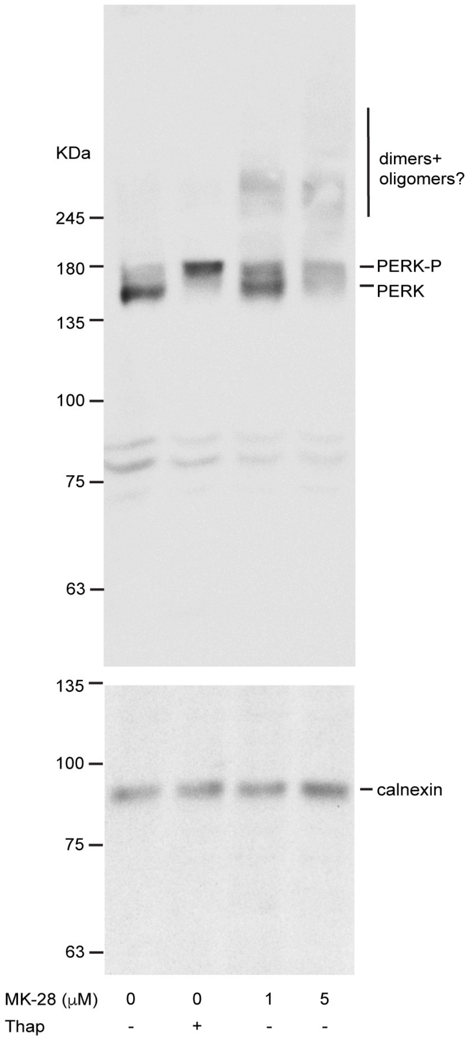


Fig. 2D:

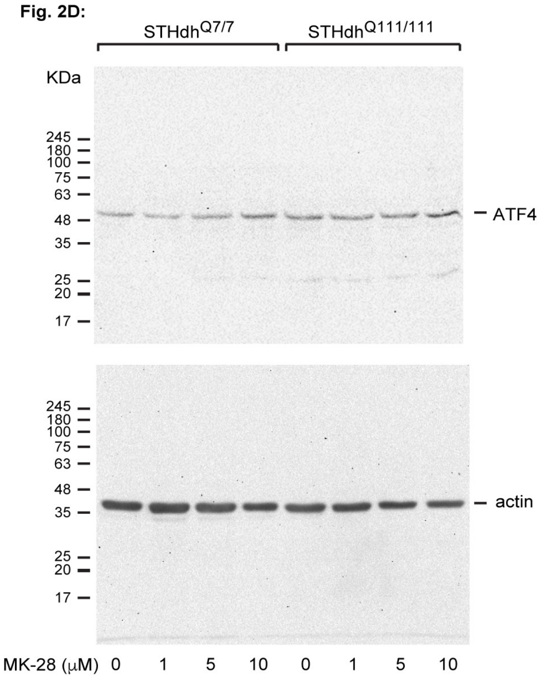


**Suppl. Fig. S6. Full length immunoblots corresponding to Fig. 2**.

**Suppl. Table S1. MK-28 full kinase panel.** MK-28 compound tested against 391 kinases. The compound was tested at a single dose in duplicates at a concentration of 1 μM. Control Compound Staurosporine was tested in 10-dose IC50 mode with 4-fold serial dilution starting at 20 or 100 μM. Alternate Control Compounds were tested in 10-dose IC50 mode with 3- or 4-fold serial dilution starting at 10, 20, or 100 μM. Reactions were carried out with 1 μM ATP.
